# Anthocyanins and musculoskeletal diseases: mechanisms and therapeutic potential

**DOI:** 10.3389/fnut.2025.1602034

**Published:** 2025-08-21

**Authors:** Xing Lv, Xiao-peng Zhao, Wen-cong Li, Nai-fei Xing, Ke-qiang Zong, Yi Zhai, Sheng-lei Yang, Ji-yao Zhang, Xia Liu

**Affiliations:** ^1^Department of Rehabilitation, Yantai Affiliated Hospital of Binzhou Medical University, Yantai, China; ^2^College of Pharmacy, Xinjiang Medical University, Ürümqi, China; ^3^College of Exercise and Health, Shenyang Sport University, Shenyang, China; ^4^Department of Neurology, Binzhou Medical University Hospital, Binzhou, China; ^5^College of Physical Education, Qiqihar University, Qiqihar, China; ^6^School of Sports Medicine and Rehabilitation, Beijing Sport University, Beijing, China; ^7^Shandong Boaoke Biotechnology Co., Ltd., Liaocheng, China; ^8^Medical Integration and Practice Center, Shandong University, Jinan, China

**Keywords:** anthocyanins, osteoporosis, rheumatoid arthritis, osteoarthritis, osteosarcoma, inflammatory, oxidative stress

## Abstract

**Background:**

Musculoskeletal diseases (MSDs) are a common group of conditions involving bones, muscles, cartilage, ligaments, and nerves, which significantly impact patients’ quality of life and ability to participate in society. Anthocyanins (ACNs), as phytochemicals, possess various pharmacological and biological activities, including anti-apoptotic, antioxidant, anti-inflammatory, and immunosuppressive properties. In recent years, ACNs have shown remarkable potential in improving MSDs. This review article aims to recapitulate the therapeutic potential of ACNs and its mechanism of action in treating MSDs.

**Methods:**

Extensive literature was searched and reviewed through online electronic databases (PubMed, Embase, and Web of Science), focusing on analysing the specific roles and molecular mechanisms of ACNs in *in vivo* and *in vitro* studies.

**Results:**

ACNs exert protective effects on MSDs by targeting multiple key signaling pathways, including mitogen-activated protein kinase (MAPK), nuclear factor-kappaB (NF-κB), Wingless-related integration site (Wnt)/β-catenin, phosphatidylinositol 3-kinase/protein kinase B (PI3K/Akt), adenosyl monophosphate-dependent protein kinase (AMPK), receptor activator of nuclear factor-kappaΒ/receptor activator of nuclear factor-kappaB ligand/osteoprotegerin (RANK/RANKL/OPG) and oxidative stress signaling. In addition, ACNs exhibited anti-inflammatory, anti-apoptotic, and immunosuppressive properties. This article reviews the mechanisms and potential therapeutic applications of ACNs in the prevention and alleviation of MSDs, providing valuable reference points for further research and development of ACNs.

**Conclusion:**

ACNs improve the prevention of MSDs through multiple actions such as antioxidant, anti-inflammatory, immunomodulatory and bone metabolism homeostasis regulation. However, results from *in vitro* and *in vivo* studies still need to be further validated by human clinical trials.

## 1 Introduction

Musculoskeletal disorders (MSDs) are a group of common disorders affecting the body’s supporting structures such as bones, muscles, tendons, cartilage, ligaments, and nerves, which are characterized by injury or dysfunction (1). These disorders are characterized by chronic pain and limitation of movement, leading to temporary or permanent loss of function, with serious adverse effects on an individual’s quality of life and social participation (2, 3). The disease burden of MSDs is increasing with the global trend of aging. It is projected that by 2050, 2 billion people worldwide will be aged 60 years or older, and more than half of these older adults will suffer from multiple chronic diseases (4, 5). Among these chronic diseases, MSDs are recognized as one of the most globally burdensome to individuals, health and social care systems (6). Hence, against the backdrop of an increasingly aging population, MSDs—characterized by their ability to cause long-term disability and dysfunction—undermine individuals’ capacity to participate in society and the workforce, further exacerbating social and economic pressures. Common MSDs include osteoporosis (OP), rheumatoid arthritis (RA), osteoarthritis (OA), and osteosarcoma (OS). Treatments for MSDs include non-pharmacological therapies (such as exercise therapy, manual therapy, and psychosocial interventions), complementary therapies (such as acupuncture), and pharmacological treatments [such as analgesics, nonsteroidal anti-inflammatory drugs (NSAIDs), and corticosteroid injections] (7–9). Although NSAIDs or opioid medications can alleviate pain, concerns about their safety and long-term efficacy limit their therapeutic effectiveness (10). Therefore, exploring safer and more effective interventions is critical for the treatment of MSDs.

With the rapid development of modern medicine, phytochemicals continue to play a vital role in disease prevention and treatment, particularly in areas such as chronic diseases, metabolic disorders, and inflammatory conditions, where they demonstrate unique advantages. Compared to synthetic drugs, phytochemicals exhibit lower toxicity and fewer side effects due to their higher biocompatibility with human tissues (11). The long-term use of synthetic drugs often leads to the development of drug resistance, especially in the case of antibiotics and antiviral medications, which has become a global health challenge (12). Anthocyanins (ACNs) are a class of natural water-soluble pigments widely found in the flowers, fruits, leaves, stems, and roots of plants, imparting vibrant red, purple, and blue colors to fruits, petals, and leaves (13). ACNs offer a wide range of health benefits in pharmacology, including antibacterial effects (14), cardioprotection (15–17), neuroprotection (18), antidiabetic effects (19, 20), anti-obesity properties (21), anti-aging effects (22), anticancer activity (23, 24), anti-inflammatory properties (5, 25), and antioxidant effects (26). Therefore, research and application of natural plant components such as ACNs show promising prospects for the future.

**Figure 1:**
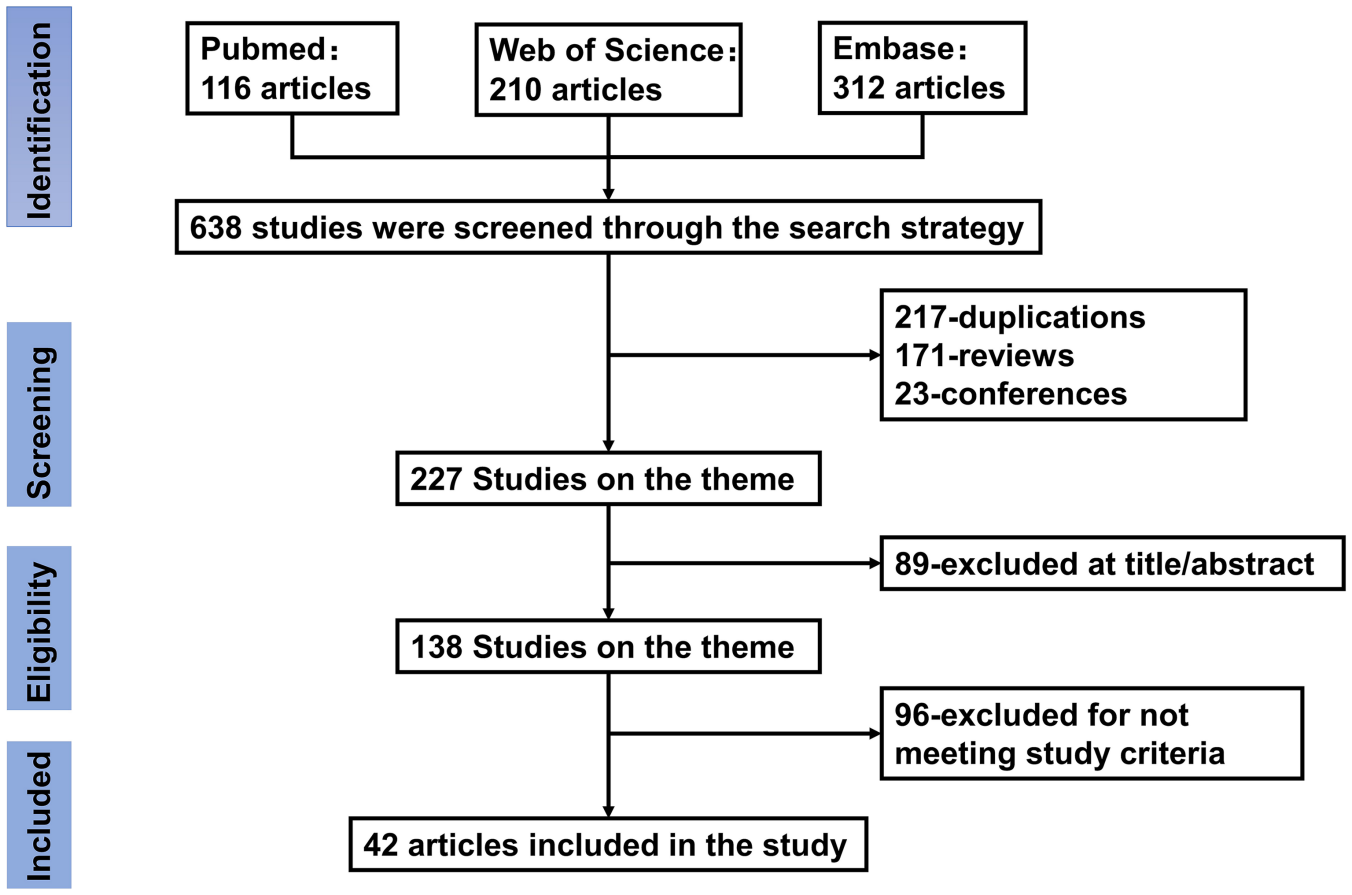
Flow chart of literature search and study selection. Initial searches retrieved 638 records from PubMed (*n* = 116), Web of Science (*n* = 210), and Embase (*n* = 312). After removing 217 duplicates, 421 records underwent title/abstract screening. Studies were excluded if they were reviews (*n* = 171) or conference abstracts (*n* = 23), leaving 227 full-text articles for eligibility assessment. A further 89 articles were excluded based on title/abstract irrelevance, and 96 articles were excluded for not meeting predefined inclusion criteria (e.g., incomplete data or off-topic content). Ultimately, 42 articles were included in the final analysis.

The system comprehensively summarized the specific therapeutic effects of ACNs in the treatment of MSDs and thoroughly explored their mechanisms of action. The aim was to provide a solid theoretical foundation and practical guidance for the potential clinical application of ACNs as an intervention for MSDs. A PRISMA flow diagram in the Figure 1 outlines the study selection process. *In vivo* and *in vitro* experiments involving anthocyanin interventions for OP, RA, OA, and OS were retrieved from the PubMed, Embase, and Web of Science databases from their inception until September 30, 2024. The keywords used were “Anthocyanins OR Cyanidin OR Malvidin OR Peonidin OR Delphinidin OR Pelargonidin OR Petunidin” AND “osteoporosis OR osteoblast OR osteoclast OR osteoarthritis OR rheumatoid arthritis OR osteosarcoma.”

## 2 Overview of anthocyanins

**Figure 2:**
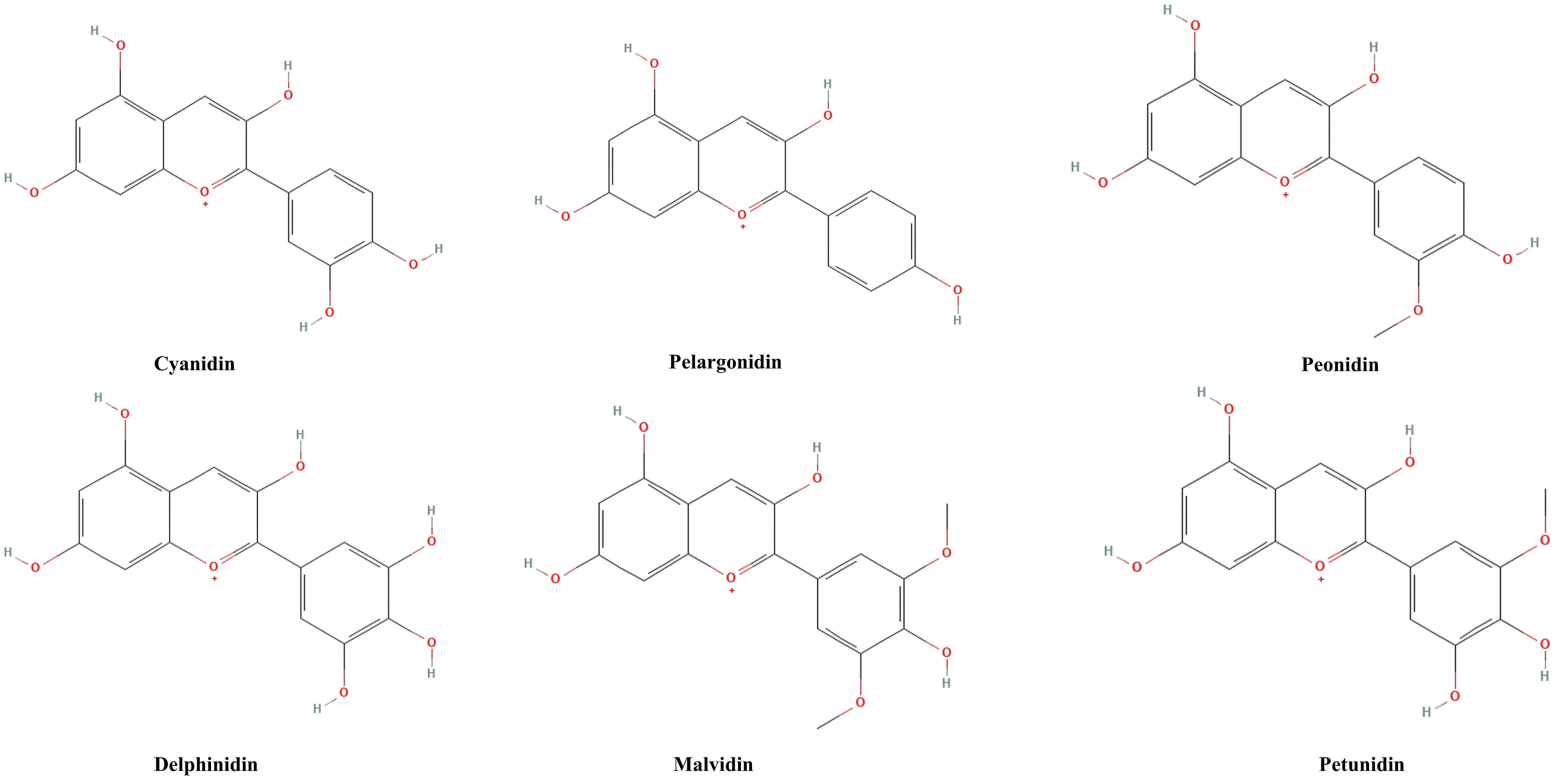
Chemical structures of six major anthocyanidins. Cyanidin, pelargonidin, peonidin, delphinidin, malvidin, and petunidin are the six main anthocyanins, which are distinguished from each other by the position and number of methoxy and hydroxyl groups in the structure of the flavylium ion.

ACNs, as natural pigments, have a history that dates back to ancient times. Humans have long extracted pigments from plants rich in ACNs for dye production and herbal formulations, while gradually recognizing their potential health benefits (27). ACNs are widely present in berry fruits (such as blueberries, cranberries, blackberries, etc.), purple sweet potatoes, pomegranates, eggplants, and grapes (28). More than 600 types of ACNs have been extracted from plants, demonstrating strong antioxidant capabilities and health benefits (29). Anthocyanin is one of the subclasses of phenolic phytochemicals. Their basic structure consists of a C₆-C₃-C₆ carbon skeleton, composed of two aromatic rings (A ring and B ring) connected by a pyran ring (C ring) (30). The fundamental unit of ACNs is called anthocyanidin, which is the aglycone part responsible for the basic chemical and biological activities of ACNs (31). Anthocyanidins are categorized into three structural classes: 3-hydroxyanthocyanidins, 3-deoxyanthocyanidins, and O-methylated anthocyanidins. In contrast, anthocyanins encompass both anthocyanidin glycosides and acylated anthocyanins (further modified by esterification with organic acids). When a glycosyl group is added to anthocyanidin, the glycosylated form of ACNs is formed. Glycosylation not only enhances the molecule’s water solubility and stability but also, to some extent, determines its color and stabilit (30). he diversity of ACNs arises not only from variations in the aglycone structures but also from the types and numbers of glycosyl groups attached to the aglycone, as well as the types and numbers of side chains on these glycosyl groups (30). These combinations lead to rich structural diversity. The six major anthocyanidins are cyanidin (Cy), pelargonidin (Pg), peonidin (Pn), delphinidin (Dp), malvidin (Mv), and petunidin (Pt) (32, 33). Figure 2 shows the base structure of six major anthocyanins. Their chemical structures enable them to act as powerful antioxidants, capable of neutralizing excess free radicals in the body and reducing oxidative stress responses (34).

ACNs possess the characteristic of exhibiting different colors depending on the environmental potential of hydrogen (pH), which imparts rich hues to plants but also makes ACNs susceptible to degradation under varying environmental conditions (35). Several factors influence the stability of ACNs, including pH, temperature, sugars (both acylated and unacylated), enzymatic activity, light exposure, and storage methods (36). Understanding the mechanisms by which these factors affect the stability of ACNs is crucial for optimizing processing and storage conditions. Most ACNs are more stable in acidic environments, and degradation of ACNs occurs when the pH is >7 (37). Secondly, temperature is also a key factor in improving stability and preservation, and high temperature conditions accelerate their degradation (38). Prolonged exposure to light causes photodegradation of ACNs, which affects their color, antioxidant activity, and molecular stability (39). In addition, acylation treatment enhances the stability of ACNs by changing the internal structure of the molecule to protect it from water molecule attack and avoid degradation (40). Therefore, ACNs-rich products should be stored away from high temperatures, strong light, and alkaline environments. The use of acylation and the selection of appropriate packaging materials can also further extend their shelf life. These optimization measures are important for improving the stability of ACNs and maintaining their functional properties.

After being ingested, ACNs undergo different changes in pH environmental conditions, leading to structural changes. Previous studies have found that assessment of plasma levels after ingestion of ACNs-rich foods revealed low bioavailability of ACNs (41). Upon ingestion of anthocyanin-rich foods, ACNs are first absorbed or hydrolyzed in the oral cavity with the aid of salivary enzymes (42). Subsequently, most ACNs enter the stomach, where the pH conditions are favorable for anthocyanin stability. A portion of ACNs can be directly absorbed into the bloodstream in the stomach and then excreted into bile and urine (43, 44). The vast majority of ACNs then enter the small intestine, where, in the alkaline environment, structural changes occur, significantly decreasing anthocyanin stability and producing degradation metabolites. These metabolites are also absorbed by the liver and subsequently distributed to other tissues (44). Unabsorbed ACNs continue into the colon, where they are subjected to the gut microbiota environment. Gut microorganisms hydrolyze the glycosidic bonds of ACNs, forming non-glycosylated aglycones, which are further degraded into simple phenolic acids (such as protocatechuic acid) (45). However, the stability of ACNs during digestion is highly dependent on the food matrix structure. Previous studies have found that consumption of whole foods rich in ACNs, compared to isolated anthocyanin extracts, enhances stability and bioavailability during the digestive process (46). Different components in food affect the absorption and stability of ACNs differently. Glucose and proteins can impede the transport and absorption of ACNs (47). Therefore, the food matrix may provide a protective effect on ACNs, particularly before they enter the intestine, reducing the damage caused by digestive enzymes and digestive fluids, thereby enhancing the stability of ACNs before reaching the intestine. In addition to relying on the food matrix, encapsulating ACNs in microcapsules made from soy protein isolate can also extend their stability during digestion (48). Therefore, technologies aimed at fully realizing the effects of ACNs by improving their stability and bioavailability, such as optimizing food matrix combinations and developing delivery systems with enhanced stability, are important directions for future research.

Anthocyanins, as nutritional and health-promoting components, hold great potential for application, with their various health-promoting effects receiving widespread attention. Numerous *in vitro* experiments, animal models, and human studies have evaluated the biological and pharmacological potential of these molecules, confirming their ability to alleviate oxidative stress, exhibit antimicrobial activity, and intervene in the onset and progression of various non-communicable diseases (including neurodegenerative diseases and metabolic syndrome) (49). There are significant regional differences in global anthocyanin intake: China recommends a daily intake of 50 milligrams (50), the United States has an actual intake of approximately 12.5 milligrams per day (51), Europe has 19–65 milligrams per day for men and 18–44 milligrams per day for women (52), Australia has approximately 24 milligrams per day (53), and Finland can reach 150 milligrams per day (54). Although the European Union has approved anthocyanins as a food additive (E163), the European Food Safety Authority (EFSA) Scientific Panel has not yet established an acceptable daily intake (ADI) due to insufficient toxicological data. It is worth noting that no clear reports of adverse effects from anthocyanin derivatives have been found in the existing literature. Providing daily doses of dietary anthocyanins exceeding 80 mg to healthy men and women can improve certain oxidative stress and inflammatory markers, and even under high-dose intake conditions, they exhibit good tolerability (55, 56), making them an ideal candidate component for the prevention or treatment of various diseases.

## 3 Anthocyanins and musculoskeletal diseases

**Figure 3:**
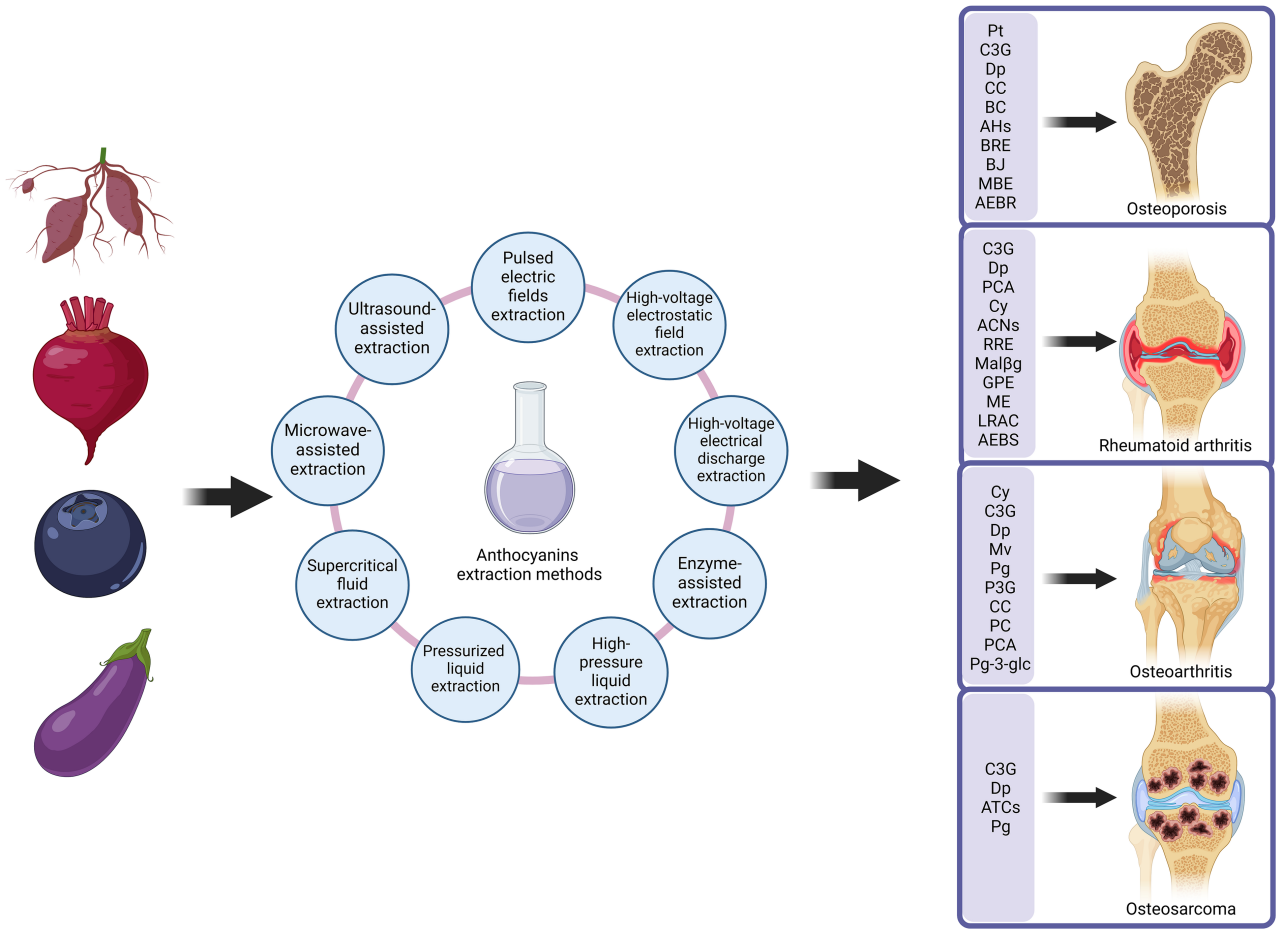
Anthocyanins improve musculoskeletal disorders. Anthocyanins are extracted from anthocyanin-rich plants in a number of ways. Extracted anthocyanins are used in the treatment of a variety of musculoskeletal disorders, including osteoporosis, rheumatoid arthritis, osteoarthritis and osteosarcoma. Created with BioRender.com.

ACNs, due to their outstanding antioxidant, anti-inflammatory, and immunomodulatory biological properties, have demonstrated significant potential in improving MSDs on Figure 3. MSDs are common health issues worldwide, primarily including OP, RA, OA, and OS. With ongoing research advancements, ACNs are gradually becoming a novel adjunctive treatment for MSDs. In the following sections, we will provide a detailed discussion of the specific mechanisms of action of ACNs in various MSDs and their application prospects, integrating the latest research findings to offer a theoretical foundation for further basic research and clinical applications.

### 3.1 Anthocyanins and osteoporosis

#### 3.1.1 Overview of osteoporosis

OP is a systemic skeletal disease characterized by low bone mass, deterioration of bone microarchitecture, increased bone fragility, and an elevated risk of fractures (57). OP is categorized into two main types: primary and secondary OP. Primary OP primarily includes postmenopausal OP in middle-aged and elderly women and age-related OP (58, 59). Secondary OP is induced by diseases or medications (60). Moreover, the core pathological mechanism of OP involves an imbalance between increased bone resorption and decreased bone formation. The receptor activator of nuclear factor-kappaΒ (NF-κB) ligand (RANKL)/receptor activator of nuclear factor kappa-Β (RANK)/osteoprotegerin (OPG) pathway plays a crucial role by regulating osteoclast activity, and estrogen deficiency further exacerbates bone loss (61, 62). Genetic factors, nutritional deficiencies, and inflammatory responses also significantly contribute to the development of OP (62). Therefore, common OP animal models are induced by ovariectomy, steroid hormones, and aging, corresponding to primary and secondary OP in humans.

#### 3.1.2 Effects of anthocyanins on osteoporosis animal studies

OP animal models are primarily categorized into primary and secondary types. This section first introduces the role of ACNs in primary OP animal models, particularly focusing on the ovariectomized (OVX) rat model and aged rat models, along with their potential mechanisms. In the OVX rat model, studies have demonstrated that ACNs exhibit significant bone-protective effects within a specific dosage range. Nagaoka et al. (63) and Kaume et al. (64) found that maqui berry extract (MBE) and cyanidin 3-O-b-d-glucoside (C3G)-rich blackberries (BBs) exerted dose-dependent bone-protective effects in OVX rats. Through computed tomography (CT) and bone histomorphological analyses, it was observed that MBE and BBs significantly increased the trabecular thickness (Tb.Th) and number (Tb.N), bone volume fraction (BV/TV), and node to terminus ratio (N.Nd/N.Tm) of the femur and tibia, while decreasing trabecular separation (Tb.Sp) (63, 64). Additionally, the results of fluorescent staining with calcein further indicated that MBE was able to promote bone mineralization (63). However, the osteoprotective effect of C3G did not show a positive effect at all doses. Kaume et al. (64) found that at high doses, C3G-rich BBs did not produce a significant protective effect on tibia, and even led to a significant decrease in the BV/TV of vertebrae and caused a deterioration of the trabecular structure model index (SMI). This phenomenon may be related to the fact that C3G-rich BBs has an extremely high oxygen radical absorbance capacity (ORAC), which may not be effective in alleviating oxidative stress because of the high dose. It may also be related to the fact that the high fiber content in high-dose BB inhibits the absorption of minerals essential for bone formation, such as calcium and phosphorus. In addition, other studies have further validated similar findings, such as Dp, and anthocyanin-rich extract from black rice (AEBR) improving bone microarchitecture in OVX rats with dose-dependent effects (65, 66). Taken together, these studies suggest that ACNs can improve the spatial connectivity and density of the trabecular network by inhibiting bone resorption and promoting bone formation, thereby enhancing bone strength and stability. Nevertheless, it is important to note that not all ACNs yield consistent effects. For example, one study reported that after oral administration of *Vaccinium myrtillus* extract (VME) at 500 mg/kg for 8 weeks in OVX rats, no improvements were observed in bone histomorphological analyses (67). This outcome might be related to the use of three-month-old OVX rats in the study. Although three-month-old rats are considered “mature,” their bone metabolism remains dynamically changing, which may obscure the bone-protective effects of blueberry extract. The specificity of the active components and the complexity of the extract composition in VME could also influence the results. Despite containing Cy and Dp, the quantities and ratios of these components in VME might be insufficient to exert significant effects. Moreover, the specific composition of ACNs may be more critical than their total content, and such compositional differences could be key factors leading to varying efficacy.

In the following, we will focus on the role and potential mechanisms of ACNs in age-related OP animal models. Sakaki et al. (68) investigated the protective effects of blackcurrant (BC) on age-related bone loss. In young mice, BC significantly increased BV/TV and Tb.N, while Tb.Sp. It also significantly decreased the levels of the bone resorption marker C-terminal telopeptide of type I collagen (CTX-1) and increased the activity of glutathione peroxidase (GPx). These findings suggest that BC protects bone mass by inhibiting bone resorption and alleviating oxidative stress. However, in aged mice, although BC slightly increased the activity of antioxidant enzymes such as catalase (CAT) and reduced the levels of the pro-inflammatory factor tumor necrosis factor-alpha (TNF-α), it did not significantly improve BV/TV or other skeletal parameters (68). The TNF-α concentration of aged mice consuming the control diet in the study increased with age, whereas BC intake had no effect on TNF-α concentration in young mice, which may be due to the fact that the anti-inflammatory effect of BC is more pronounced in the pro-inflammatory state. The lack of response to BC intervention in older mice may be related to the fact that bone loss is already severe at their older age, and the bone trabeculae in older mice may have been too fragile to achieve effective repair before BC dietary intervention (69). Thus, BC is a potential dietary supplement for ameliorating bone loss, but its effects are more pronounced in younger individuals and its efficacy is limited in older individuals.

Next, the role and potential mechanisms of ACNs in animal models of drug-induced OP will be further explored. Karunarathne et al. (70) demonstrated the role of anthocyanin-enriched polyphenols from *Hibiscus syriacus* L. (AHs) in resisting prednisolone (PDS)-induced OP. PDS is a common glucocorticoid, and its long-term use inhibits bone formation and increases bone resorption, thereby causing a decrease in bone mineral density (BMD). Studies have shown that calcein staining analysis by calcein staining showed that AHs significantly elevated the number of vertebrae and the strength of calcification in PDS-induced bone mineral density in mice, improving the decline in BMD (70). In addition, ACNs have been shown to have significant effects in ameliorating bone loss in animal models of diabetes. Diabetes-induced bone loss is mainly associated with disturbances in bone metabolism due to chronic hyperglycemia, such as increased oxidative stress, impaired bone mineralization, and enhanced bone resorption. A study in a streptozotocin (STZ)-induced diabetic rat model found that AEBR played a significant role in preserving the structure of bone tissue and regulating metabolic disturbances (71). Specifically, the bone histology of diabetic rats was significantly improved by oral administration of AEBR for 8 consecutive weeks. Dual-energy X-ray absorptiometry (DEXA) analysis showed that AEBR treatment significantly increased lumbar spine and femur BMD in diabetic rats. Further morphometric analyses of bone histology showed that AEBR was able to increase BV/TV, Tb.Th, and cortical thickness (Ct.Th), and decreased Tb.Sp. These osteoprotective effects were accompanied by metabolic improvements, including dose-dependent decreases in blood glucose levels and RANKL concentrations, and increases in calcium (Ca) and phosphate (P) concentrations (71). These results suggest that AEBR not only protects bone by improving trabecular microarchitecture, but also plays an indirect role by modulating metabolic disorders. Osteoclast overactivity is a central mechanism in many OP models, and ACNs have shown unique potential in regulating osteoclast activity. For example, Moriwaki et al. (65) showed that Dp was able to slow down bone loss by inhibiting osteoclast activity. In soluble RANKL (sRANKL)-induced OP model mice, 14 days of gavage of Dp significantly improved femoral Tb.Th, BV/TV, and Tb.N (65). Similarly, two studies by Nagaoka et al. (63, 72) found that MBE and Pt also exhibited dose-dependent osteoprotective effects in the sRANKL-induced mouse model of OP. These studies suggest that ACNs may protect bone by inhibiting the RANKL signaling pathway and reducing osteoclast production and activity. In addition, inflammation plays an important role in some OP pathologies, such as the lipopolysaccharide (LPS)-induced inflammatory bone loss model. LPS can stimulate osteoclast activity by promoting the release of inflammatory factors, thereby exacerbating bone resorption (73). Park et al. (74) found that protocatechuic acid (PCA) could significantly improve bone tissue parameters and reduce inflammation-induced bone loss by inhibiting LPS-induced inflammatory responses. This further supports the potential of ACNs in modulating skeletal inflammatory responses. In summary, AHs, AEBR, Dp, PCA, MBE, and Pt exhibited significant osteoprotective effects in animal models of OP induced by various drugs (PDS, STZ, RANKL, and LPS). These their mechanisms include promoting BMD enhancement, improving bone trabecular microarchitecture, modulating metabolic disorders and inhibiting inflammation-induced bone loss.

#### 3.1.3 Molecular actions of anthocyanins in the preventing osteoporosis

##### 3.1.3.1 The regulation of osteogenesis via Runx2

Runt-related transcription factor 2 (Runx2) is a key transcription factor in the process of bone formation, which mainly regulates osteoblast differentiation, expression of bone matrix proteins, and formation and reconstruction of bone tissue. Runx2 plays a critical role in the early stages of osteoblast differentiation, guiding mesenchymal stem cells (MSCs) through the activation of osteogenic-specific genes such as osteocalcin (OCN), osteopontin (OPN), and bone sialoprotein (BSP) (75). Runx2 deficiency hinders the differentiation of MSCs, resulting in impaired bone formation. In addition, Runx2 regulates the expression of bone matrix proteins and mineralization-related enzymes by binding to the OSE2 site on the promoter of the OCN gene, thereby affecting the mineralization process of bone tissue (76). Notably, the function of Runx2 is not limited to a single signaling pathway, as its expression and activity are regulated by multiple signaling molecules. For example, bone morphogenetic proteins (BMPs) upregulate Runx2 expression through the small mothers against decapentaplegic (Smad) signaling pathway (77), while the Wingless-related integration site (Wnt)/β-catenin signaling pathway enhances Runx2’s transcriptional activity on osteogenic genes (78), further promoting osteoblast maturation. With advancing research, increasing evidence suggests that ACNs—such as MBE, AEBR, AHs, blueberry juice (BJ), and C3G—play a significant role in bone formation by regulating the expression and function of Runx2. Nagaoka et al. (63) found that MBE upregulates the expression of the key osteogenic transcription factor Runx2 and its upstream regulator BMP2, while also promoting the expression of downstream genes such as Osx, Ocn, and Mepe, thereby increasing mineralized nodule formation and alkaline phosphatase (ALP) activity. This indicates that MBE promotes osteoblast differentiation and mineralization through the BMP2-Runx2 axis. Furthermore, studies on AEBR in diabetic models have shown that it can restore Runx2 expression in bone tissue and serum, thereby improving osteogenic dysfunction caused by diabetes (71).

Furthermore, Runx2 and the Wnt/β-catenin signaling pathway jointly regulate the expression of osteoblast-specific genes, thereby promoting osteoblast differentiation and bone matrix mineralization (79). Karunarathne et al. (70) found that AHs promote the phosphorylation of glycogen synthase kinase-3 beta (GSK-3β) at the SER9 site, inhibiting its activity and allowing β-catenin to translocate into the nucleus, where it activates the transcription of osteoblast-specific genes, including Runx2. Moreover, sirtuin type 1 deacetylase (Sirt1) increases the transcriptional activity of Runx2 through deacetylation, thereby promoting the expression of osteogenesis-related genes (80). Domazetovic et al. (81) also found that BJ enhances the expression and functional activation of Runx2 and ALP by activating Sirt1-mediated deacetylation, thereby improving osteoblast differentiation and mineralization (81). However, the regulatory effects of C3G on Runx2 vary across experiments. Hu et al. (82) found that C3G significantly increased the expression of ALP, Runx2, and OCN, demonstrating that OCN protein expression is regulated by extracellular signal-regulated kinase (ERK), while ALP and Runx2 expression are not affected by ERK. Park et al. (83) found that C3G treatment did not alter the expression levels of collagen type I alpha 1 chain (Col1α1) and Runx2 but promoted bone formation by enhancing the expression of downstream osteogenic differentiation marker genes (OPN, OCN, ALP) and the key transcription factor Osx. The expression of Osx can be regulated through both Runx2-dependent and Runx2-independent signaling pathways (84). Therefore, the stimulatory effect of C3G on osteoblasts may be Runx2-independent, and C3G-induced Osx expression does not affect Col1α1 expression. In summary, MBE, AEBR, AHs, BJ, and C3G regulate the expression and functional activity of Runx2 through multiple signaling pathways, including upstream BMP2 signaling, Wnt/β-catenin, oxidative stress modulation, and ERK1/2 phosphorylation.

##### 3.1.3.2 The regulation of osteoclastogenesis via OPG and RANKL

RANKL is a core factor regulating the generation, activation, and functional maintenance of osteoclasts. By binding to the RANK receptor on the surface of osteoclasts and their precursor cells, RANKL activates a series of signaling pathways, such as NF-κB and mitogen-activated protein kinase (MAPK), promoting osteoclast activation and bone resorption (85, 86). In contrast, osteoprotegerin (OPG), a soluble receptor protein secreted by osteoblasts, competitively binds to RANKL, preventing its interaction with RANK, thereby inhibiting osteoclast generation and activation and suppressing bone resorption (87, 88). The OPG/RANKL ratio is crucial for maintaining the balance between bone formation and bone resorption (89). In bone metabolic diseases, particularly diabetic OP, the imbalance in RANKL and OPG expression is particularly pronounced. Hyperglycemia and oxidative stress in diabetic conditions stimulate RANKL expression while suppressing OPG expression, disrupting the OPG/RANKL balance (90), Qi et al. (71) found that AEBR effectively reduced the overexpression of RANKL in diabetic rats, ultimately restoring the OPG/RANKL ratio and thereby inhibiting excessive osteoclast activity. AEBR also enhanced osteoblast differentiation by promoting Runx2 expression, thus promoting bone formation while reducing bone resorption (71). This dual regulatory effect indicates that AEBR has comprehensive protective effects in bone metabolic balance. However, not all ACNs can effectively regulate the OPG/RANKL system. Casati et al. (91) found that delphinidin-3-rutinoside (D3R) failed to alter the increased RANKL expression and decreased OPG expression induced by tert-butyl hydroperoxide (t-BHP). This may be due to the glycosylation modification on D3R (i.e., rutinose at the 3rd position), which could affect its bioactivity, leading to its differential effects on osteoclastogenesis.

Nuclear factor of activated T cells calcineurin-dependent 1 (NFATc1) is a key transcription factor in osteoclastogenesis and a critical component of the RANKL-induced signaling pathway (92–94). Specifically, NFATc1 is the main transcription factor located at the convergence point downstream of the NF-κB and c-Fos signaling pathways during osteoclastogenesis (94). The expression and activation of NFATc1 further regulate the expression of many osteoclast-specific genes, such as dendritic cell-specific transmembrane protein (DC-STAMP), osteoclast stimulatory transmembrane protein (OC-STAMP), calcitonin receptor (CTR), and cathepsin K (CtsK) (92, 93, 95). In recent years, multiple studies have found that MBE, Pt, cyanidin chloride (CC), C3G, Dp, and protocatechuic acid (PCA) can inhibit osteoclast differentiation and generation by downregulating RANKL-induced c-Fos and NFATc1 expression, thereby suppressing the expression of downstream genes (DC-STAMP, OC-STAMP, CTR, and CtsK) (63, 65, 72, 74, 83, 96). These effects suggest that ACNs may intervene in the RANKL signaling pathway and inhibit osteoclast-associated pathological processes by targeting NFATc1, a key transcription factor in osteoclastogenesis. Notably, the regulatory effects of different ACNs on NFATc1 may involve different signaling pathways. Park et al. (83) found that C3G inhibits RANKL-mediated osteoclastogenesis by suppressing MAPK activation, thereby downregulating c-Fos and NFATc1 expression. Meanwhile, Cheng et al. (96) provided another mechanistic explanation from the perspective of calcium signaling. Under CC treatment, RANKL-mediated calcium oscillations were significantly reduced, thereby inhibiting the transcriptional activity of NFATc1 (96). Ca oscillations are a critical step in the RANKL signaling pathway for NFATc1 activation, as calcium signaling activates calcineurin, leading to the dephosphorylation and nuclear translocation of NFATc1, thereby initiating transcription (97). This finding indicates that CC can influence NFATc1 activation by interfering with the calcium signaling pathway, further elucidating the molecular mechanisms of ACNs in osteoclastogenesis.

RANKL promotes the differentiation of osteoclast precursor cells into mature osteoclasts by activating the NF-κB signaling pathway, a critical step in bone resorption. The activation of the NF-κB signaling pathway depends on the binding of RANKL to the RANK receptor on the surface of osteoclast precursors, which subsequently induces the phosphorylation and degradation of IκBα, allowing the p65 subunit of NF-κB to translocate into the nucleus and initiate the expression of genes related to osteoclast differentiation and function (85, 86). Therefore, inhibiting the RANKL-induced NF-κB signaling pathway is considered a potential therapeutic strategy to reduce osteoclastogenesis and bone resorption. Multiple studies have found that Pt, CC, and Dp significantly reduce osteoclast generation and bone resorption by inhibiting RANKL-induced nuclear translocation of NF-κB p65, thereby suppressing the NF-κB signaling pathway (65, 72, 96). However, not all ACNs regulate the NF-κB signaling pathway. One study showed that C3G does not significantly inhibit the activation of IκBα or the NF-κB signaling pathway (83). This result suggests that the molecular structure of different ACNs may lead to their differential regulation of the NF-κB pathway. C3G failed to significantly inhibit the NF-κB pathway, but it indirectly affects osteoclastogenesis and function by inhibiting RANKL signaling through inhibition of the MAPK pathway. Therefore, the specific mechanism of action of different types of ACNs needs to be further investigated. In summary, Pt, Dp, and CC have significant effects on the prevention and treatment of OP by inhibiting RANKL-induced activation of the NF-κB signaling pathway and blocking osteoclast differentiation and bone resorption.

**Figure 4:**
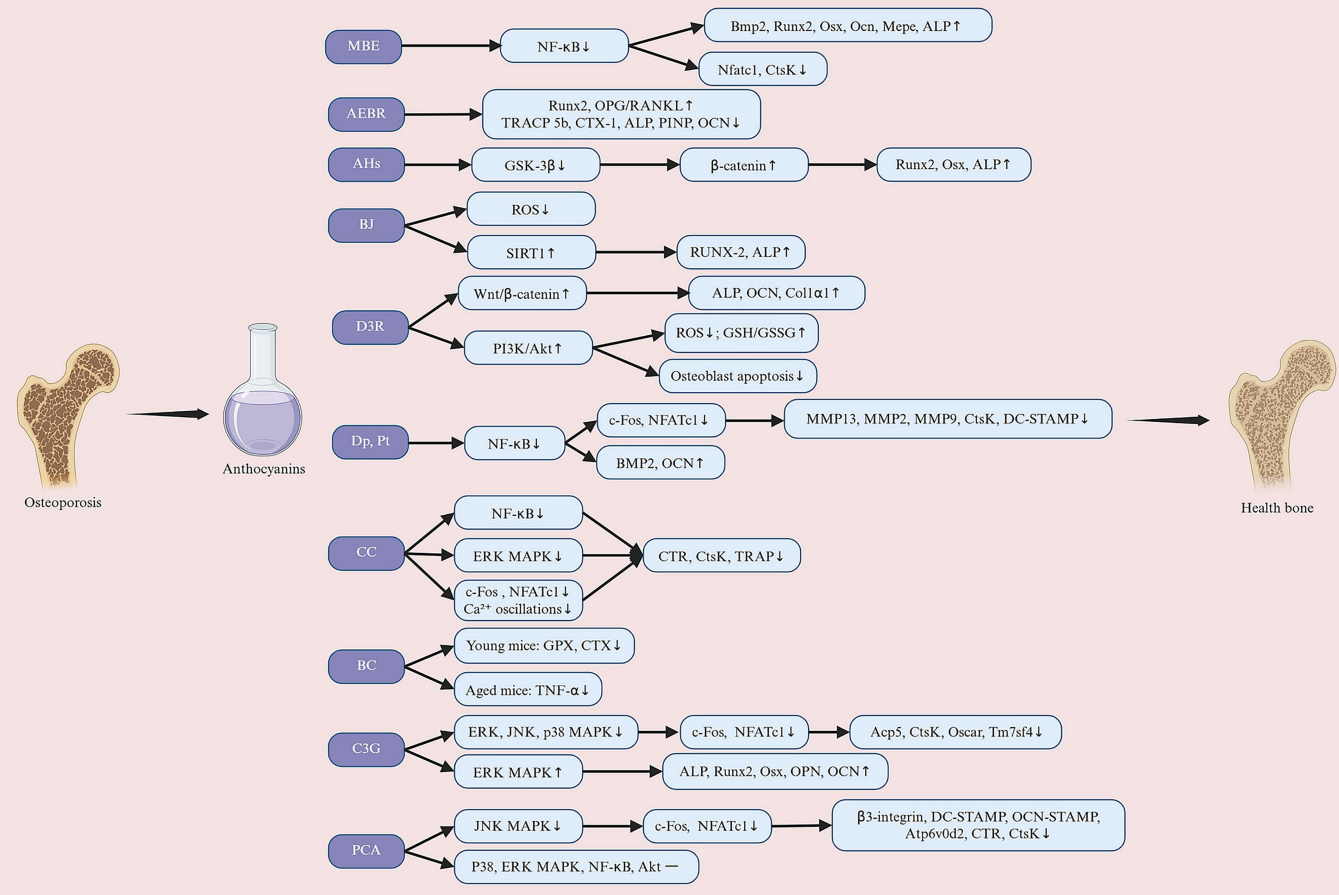
Anthocyanins exert their preventive and therapeutic effects against osteoporosis through multiple molecular mechanisms. Their pro-osteogenic effects involve promoting osteoblast differentiation, including the activation of signaling pathways such as Wnt/β-catenin, ERK/MAPK, and BMP, inhibiting GSK-3β, upregulating SIRT1 and BMP2, enhancing antioxidant capacity, and ultimately promoting the expression of Runx2, ALP, and other osteogenic-related factors, improving bone matrix mineralization levels, and increasing bone formation. Additionally, anthocyanins inhibit osteoclast activity (anti-osteoclastogenic) by suppressing NF-κB, RANKL-related signaling, and calcium signaling pathways, reducing the expression of osteoclast-related genes (such as c-Fos, NFATc1, TRAP, CTSK, and MMP9), increasing the OPG/RANKL ratio, and decreasing bone resorption. MBE, maqui berry extract; AEBR, anthocyanin-rich extract from black rice; AHs, anthocyanin-enriched polyphenols from *Hibiscus syriacus* L.; BJ, blueberry juice; D3R, delphinidin-3-rutinoside; Dp, delphinidin; Pt, petunidin; CC, cyanidin chloride; BC, blackcurrant; C3G, cyanidin-3-glucoside; PCA, protocatechuic acid. Created with BioRender.com.

The MAPK signaling pathway is a key component of RANKL signaling, and its activation is critical for osteoclast differentiation and function. Cheng et al. (96) showed that CC significantly inhibited RANKL-induced ERK1/2 phosphorylation, thereby reducing osteoclast differentiation and bone resorption capacity. Further studies have expanded the understanding of the regulation of the MAPK pathway. Park et al. (83) found that C3G significantly inhibited RANKL-induced activation of multiple MAPK family members (including ERK, JNK and p38), with the most significant effect in the inhibition of p38. Notably, p38 not only promotes the expression of c-Fos and NFATc1 during osteoclastogenesis, but also supports osteoclast function by regulating the expression of cell fusion and bone resorption-related proteins (98). Therefore, the inhibitory effects of C3G on multiple MAPK pathways may exert its effects through multiple targets, and this broad inhibition suggests that C3G may be a promising candidate compound with significant anti-resorptive potential. Nevertheless, different ACNs may have specific effects on the MAPK pathway. Park et al. (74) found that PCA treatment reduced JNK phosphorylation but did not significantly improve RANKL-induced p38 or ERK phosphorylation. This indicates that PCA may inhibit RANKL-mediated osteoclastogenesis by inactivating JNK and reducing AP-1 transcriptional activity. In summary, CC, C3G, and PCA play a positive regulatory role in bone metabolic balance by interfering with different branches of the MAPK signaling pathway, thereby significantly reducing osteoclast differentiation and bone resorption function (Figure 4).

##### 3.1.3.3 The regulation of bone metabolism via oxidative factors

Oxidative stress is a pathological state caused by the excessive generation of reactive oxygen species (ROS) and an imbalance in the antioxidant defense system, which has significant negative effects on bone metabolism (99). High levels of ROS play a dual role in bone metabolism: ROS promote the differentiation and activity of osteoclasts by activating osteoclast-related signaling pathways, thereby accelerating bone resorption and leading to bone loss. On the other hand, ROS inhibit the differentiation and function of osteoblasts and further disrupt bone formation by inducing osteoblast apoptosis (100). This imbalance between enhanced bone resorption and impaired bone formation is a major pathogenic mechanism of OP. Targeting this mechanism, studies have found that various anthocyanin compounds can mitigate the negative effects of oxidative stress on bone metabolism through multiple pathways. Casati et al. (91) discovered that D3R can significantly reduce cell apoptosis induced by tert-Butyl hydroperoxide (t-BHP), protecting MC3T3-E1 cells from oxidative stress damage. This protective effect is achieved by reducing intracellular ROS generation, increasing glutathione levels, and maintaining the glutathione/glutathione disulfide ratio, thereby enhancing antioxidant capacity. Additionally, D3R alleviates oxidative stress-induced cell damage by activating the phosphoinositide-3-kinase/protein kinase B (PI3K/Akt) pathway (91). ALP and Runx2 are hallmark molecules of osteoblast differentiation, and their activity levels directly reflect the functional state of osteoblasts. Domazetovic et al. (81) demonstrated that BJ can significantly reduce ROS levels induced by buthionine sulfoximine (BSO) and restore the activity of ALP and Runx2 associated with bone formation. This indicates that BJ partially or completely reverses the inhibitory effects of oxidative stress on bone formation by alleviating the suppression of these molecules.

With aging, the decline in sex hormone secretion further weakens the oxidative defense system, exacerbating the negative effects of oxidative stress on bone health (101). Sakaki et al. (68) demonstrated that BC significantly enhances the activity of Glutathione peroxidase (GPX) in young female mice, exhibiting strong antioxidant capacity. This occurs simultaneously with an increase in trabecular bone mass, suggesting that improved antioxidant defense may inhibit bone resorption and ultimately improve bone mass. However, this effect was not significant in aged female mice, suggesting that BC’s effects in young mice may be primarily mediated through enhancing antioxidant enzyme activity, while in aged mice, it may manifest more as an anti-inflammatory effect. Additionally, estrogen itself protects bone health by inhibiting the generation of ROS in bone tissue, and its deficiency leads to OP by reducing thiol antioxidants in osteoclasts (102, 103). The potential protective effects of ACNs against oxidative stress induced by estrogen deficiency have been further confirmed. Nagaoka et al. (63) demonstrated that MBE significantly reduces MDA levels in the plasma of OVX mice and sRANKL-induced mice. However, not all ACNs exhibit consistent effects in reducing oxidative stress in OVX mice. Kaume et al. (64) found that C3G-rich BBs at a high dose of 10% (w/w) did not significantly improve the levels of SOD, GPX, CAT, and thiobarbituric acid reactive substances (TBARS) in OVX rats. Compared to other ACNs, C3G has the highest oxygen radical absorbance capacity (104). The lack of efficacy at the 10% (w/w) dose may be due to the excessively high concentration of BB, which could not effectively counteract oxidative stress in OVX mice. Future studies could explore the effects of BB at lower concentrations, such as below 5, 5, and 7.5% (w/w), to investigate its impact on bone metabolism. In summary, these studies indicate that D3R, BJ, MBE, and BC reduce the negative effects of oxidative stress on bone metabolism by decreasing ROS generation, modulating signaling pathways such as PI3K/Akt, and enhancing the antioxidant defense system, thereby protecting bone health. The mechanisms and limitations of C3G require further in-depth investigation.

##### 3.1.3.4 The regulation of bone metabolism via inflammatory factors

Under inflammatory conditions, the levels of pro-inflammatory factors such as TNF-α and interleukin-1 beta (IL-1β) are significantly elevated. These factors exacerbate bone resorption by enhancing osteoclast differentiation and activity, thereby disrupting bone metabolic balance (105). In recent years, studies have shown that ACNs exhibit anti-inflammatory effects in animal models of primary OP through multiple mechanisms. Sakaki et al. (68) demonstrated that BC intake significantly reduces TNF-α concentrations in aged mice but has no significant effect on IL-1β levels. In young mice, BC did not significantly affect the concentrations of TNF-α or IL-1β (68). This suggests that the anti-inflammatory effects of BC are more pronounced under pro-inflammatory conditions and may be limited when inflammatory factor levels are low. This difference may be due to the concentration of BC in the study being insufficient to reduce IL-1β levels or because aging did not significantly increase IL-1β levels, potentially weakening BC’s anti-inflammatory effects on IL-1β. The effects of anthocyanins on osteoporosis in *in vivo* and *in vitro* studies are summarized in Table 1.

**Table 1:** The effects of anthocyanins on osteoporosis *in vivo* and *in vitro* studies.

Model		Treatment dose	Treatment route	Signaling pathway/Mechanisms	Intervention outcome	References
*In vivo*/*In vitro*	Animal/Cell					
*In vitro*	MC3T3-E1 cells RANKL-induced BMM	0–25 μg/mL MBE	N/A	NF-κB Oxidative stress	ALP, BMP2, Runx2, Osx, Mepe, OCN↑; BMP4, NFATc1, CtsK, MDA↓	(63)
*In vivo*	RANKL, OVX-induced C57BL/6J mice	12.5–37.5 mg/kg/day MBE for 14, 28 days	Gavage			
*In vivo*	STZ-induced diabetic rats	0.5, 1.0, 2.0 g/kg/day AEBR for 8 weeks	Gavage	N/A	OCN, ALP, PINP↓; OPG, Runx2↑ RANKL, TRACP 5b, CTX-1↓	(71)
*In vitro*	PDS-induced MC3T3-E1 cells	0-100 μg/mL AHs	N/A	Wnt/β-catenin	Osx, Runx2, ALP↑	(70)
*In vivo*	PDS-osteoporosis zebrafish larvae	0–200 μg/mL AHs for 6 days	Oral			
*In vivo*	BSO-induced SaOS-2 cells	7.5, 15 μg/mL BJ	N/A	Oxidative stress	ALP, Runx2↑; ROS↓; Sirt1↑	(81)
*In vivo*	OVX induced Sprague–Dawley mice	500 mg/kg/day VME for 8 weeks	Gavage	N/A	N/A	(67)
*In vitro*	t-BHP-induced MC3T3-E1 cells	10^−5^–10^−11^ M D3R	N/A	Wnt/β-catenin; PI3K/Akt Oxidative stress	ALP, OCN, Col1α1↑; ROS↓; GSH/GSSG↑	(91)
*In vitro*	Human primary osteoblasts MC3T3-E1 cells	0–400 μmol/L C3G	N/A	ERK	ALP, Runx2, OCN↑	(82)
*In vitro*	MC3T3-E1 cells sRANKL-induced RAW264.7 cells	10–50 μg/mL Pt	N/A	NF-κB Degradation of extracellular matrix	BMP2, OCN↑; MMP13, MMP2, MMP9, c-Fos, NFATc1, CtsK, DC-STAMP↓	(72)
*In vivo*	sRANKL-induced C57BL/6J mice	7.5 mg/kg/day Pt for 14 days	Oral			
*In vitro*	RANKL-induced BMMs	0-20 μM CC	Intraperitoneally	NF-κB; ERK	CTR, CtsK, TRAP, NFATc1, c-Fos↓	(96)
*In vivo*	OVX induced C57BL/6J mice	5 mg/kg once every 2 days CC for 6 weeks	N/A			
*In vivo*	3 months old and 18 months old C57BL/6J mice	1%w/w BC for 4 months	N/A	Oxidative stress Inflammation	Young mice: GPX, CTX↓ Aged mice: TNF-α↓	(68)
*In vitro*	RANKL, M-CSF-induced BMMs ascorbic acid and β-glycerophosphate-induced osteoblasts	100 μM C3G	N/A	ERK, JNK, p38	Acp5, CtsK, Oscar, Tm7sf4, Atp6v0d2, c-Fos, NFATc1↓; OPN, ALP, Osx↑	(83)
*In vitro*	RANKL-induced RAW264.7 cells	0.25–20 μg/mL Dp	N/A	NF-κB	MMP9, TRAP, c-Fos, NFATc1, DC-STAMP↓	(65)
*In vivo*	sRANKL, OVX-induced C57BL/6J mice	1–10 mg/kg/day Dp for 10, 28 days	Oral			
*In vitro*	M-CSF, RANKL-induced BMMs	1, 3 μg/mL BC	N/A	N/A	N/A	(66)
*In vivo*	OVX induced C57BL/6J mice	1% BC for 4-12 Weeks	N/A			
*In vitro*	RANKL-induced BMMs	250 μM PCA	N/A	JNK	c-Fos, NFATc1, β3-integrin, DC-STAMP, OCN-STAMP, Atp6v0d2, CTR, CtsK↓	(74)
*In vivo*	LPS-induced ICR mice	25 mg/kg PCA for 9 days	Oral			
*In vivo*	OVX-induced Sprague–Dawley rats	5, 10% w/w BBs for 100 days	Oral	N/A	N/A	(64)

↑, increased; ↓, decreased. RANKL, receptor activator of nuclear factor-kappaΒ ligand; BMMs, bone marrow macrophages; MBE, maqui berry extract; OVX, ovariectomized; NF-κB, nuclear factor-kappaΒ; ALP, alkaline phosphatase; BMP, bone morphogenetic protein; Osx, osterix; Mepe, matrix extracellular phosphoglycoprotein; OCN, osteocalcin; NFATc1, nuclear factor of activated T cells calcineurin-dependent 1; CtsK, cathepsin K; MDA, malondialdehyde; STZ, streptozotocin; AEBR, anthocyanin-rich extract from black rice; PINP, N-terminal propeptide of type I procollagen; OPG, osteoprotegerin; Runx2, runt-related transcription factor 2; TRACP 5b, tartrate-resistant acid phosphatase 5b; CTX-1, C-terminal telopeptide of type I collagen; PDS, prednisolone; AHs, anthocyanin-enriched polyphenols from *Hibiscus syriacus* L.; Wnt, wingless-related integration site; BSO, butionine sulfoximine; BJ, blueberry juice; Sirt1, sirtuin 1; VME, *Vaccinium myrtillus* extract; t-BHP, tert-butyl hydroperoxide; D3R, delphinidin-3-rutinoside; PI3K/Akt, phosphoinositide-3-kinase/protein kinase B; Col1α, collagen type I alpha 1 chain; C3G, cyanidin-3-glucoside; ERK, extracellular signal-regulated kinase; sRANKL, soluble RANKL; Pt, petunidin; MMP, matrix metalloproteinases; DC-STAMP, dendritic cell-specific transmembrane protein; CC, cyanidin chloride; BC, blackcurrant; GPX, Glutathione peroxidase; TNF-α, tumor necrosis factor-alpha; M-CSF, macrophage colony-stimulating factor; JNK, c-Jun N-terminal kinase; Acp5, acid phosphatase 5; Tm7sf4, transmembrane 7 subfamily member 4; Atp6v0d2, ATPase H^+^ transporting V0 subunit d2; OPN, osteopontin; Dp, delphinidin; PCA, protocatechuic acid; LPS, lipopolysaccharide; BBs, blackberrie.

### 3.2 Anthocyanins and rheumatoid arthritis

#### 3.2.1 Overview of rheumatoid arthritis

RA is an autoimmune disease characterized by chronic synovial hyperplasia, progressive symmetric joint inflammation, and autoantibody generation (106, 107). Adjuvant-induced arthritis (AIA) and collagen-induced arthritis (CIA) are the most commonly used animal models for the study of RA, and they are able to mimic the key features of RA, such as the abnormal proliferation of synovial tissues, swelling of the joints, and the destruction of cartilage and bone tissues (108). Therefore, the aim of this review is to summarize the current information about the potential efficacy of ACNs in the treatment of RA, and to analyze and discuss their mechanisms of action and findings.

#### 3.2.2 Effects of anthocyanins on rheumatoid arthritis animal studies

In recent years, several studies have explored the role of anthocyanin analogs in animal models of RA, showing their unique potential to reduce inflammation and protect joint structures. Decendit et al. (109) investigated the effect of malvidin-3-O-β-glucoside (Malβg) against arthritis in adjuvant-induced arthritis (AIA) rats. The results showed that Malβg not only significantly improved the symptoms of inflammatory malady, such as weight loss and systemic inflammatory response, in the AIA model, but also outperformed conventional hydrocortisone (HC) treatment in reducing arthritis scores (109). HC is a commonly used anti-inflammatory drug, but its long-term use may lead to significant side effects, whereas Malβg showed higher therapeutic efficacy without significant adverse effects observed. This finding suggests that Malβg may provide a new strategy to alleviate inflammation and ameliorate arthropathy through mechanisms that modulate the expression of inflammatory factors or enhance anti-inflammatory signaling. Mossalay et al. (110) demonstrated that the combination of grape polyphenol extract (GPE) and propolis (PR) was able to significantly reduce arthritic symptoms, including edema and pain, and improve systemic inflammatory malignant manifestations in AIA rats. Of particular note, low doses of GPE + PR showed more significant anti-inflammatory effects than high doses, which may suggest that the anti-inflammatory mechanism involves dose-dependent optimization effects, e.g., by modulating the balance between pro- and anti-inflammatory factors. In addition, no significant side effects were observed in the study, further supporting GPE and PR their safety (110). Similarly, several studies have found that red raspberry extract (RRE), Cy, and mulberry fruit extract (ME) are effective in protecting cartilage and bone tissue by reducing inflammatory edema, relieving pain, and decreasing inflammatory cell infiltration in AIA rats (111–113). These studies further emphasize the multiple mechanisms of ACNs in modulating synovial inflammation and blocking cartilage destruction in AIA rats. In addition, C3G, anthocyanin extracted from black soybean seed coats (AEBS) also showed potent anti-arthritic effects in CIA mice. Several studies have collectively demonstrated that C3G inhibits the continued progression of synovial inflammation by significantly alleviating injuries such as severe swelling, erythema, and joint stiffness in the hind paw of CIA mice, while inhibiting the progression of synovial inflammation (114–116). Overall, the collective results from various studies indicate that Malβg, the GPE + PR combination, RRE, C3G, and Cy exhibit significant anti-inflammatory and joint-protective effects in animal arthritis models through multiple mechanisms. These mechanisms include reducing inflammatory cachexia, inhibiting the expression of inflammatory factors, alleviating joint swelling, and mitigating synovial lesions. These anthocyanin compounds have repeatedly demonstrated superior efficacy over traditional treatments in the therapy of inflammatory joint lesions while maintaining good safety profiles.

#### 3.2.3 Molecular mechanisms of anthocyanins in the prevention of rheumatoid arthritis

##### 3.2.3.1 The amelioration of joint destruction via inflammatory factors

The pathological process of RA primarily occurs in the synovium, where the maintenance of synovial tissue inflammation depends on the dynamic interactions among immune cells, fibroblast-like synoviocytes (FLS), and osteoclasts (117). In RA synovial tissue, abnormal immune activation is a critical step in the initiation and persistence of inflammation. Antigen-presenting cells activate T lymphocytes and B lymphocytes, triggering a series of inflammatory responses centered on the release of pro-inflammatory cytokines (118). These cytokines not only sustain the inflammatory response but also directly promote the differentiation and activation of osteoclasts. Additionally, they induce RAFLSs to secrete matrix metalloproteinases (MMPs), exacerbating the degradation of the cartilage matrix. In recent years, anti-inflammatory therapeutic research targeting RAFLSs and related immune cells has demonstrated significant therapeutic potential with compounds such as C3G, Cy, *L. ruthenicum* anthocyanins (LRAC), and protocatechuic acid (PCA). Sun et al. (114) reported that C3G significantly inhibits the expression of TNF-α, IL-1β, and interleukin-6 (IL-6) in lipopolysaccharide (LPS)-induced FLSs. C3G also significantly reduces serum levels of these pro-inflammatory factors in a CIA mice model, indicating its potent anti-inflammatory effects both *in vitro* and *in vivo* (114). Wang et al. (115) further elucidated that C3G alleviates inflammation and inhibits FLS proliferation by upregulating the proportion of regulatory T (Treg) cells, downregulating the proportion of CD38^+^ natural killer (NK) cells, and regulating sirtuin 6 (Sirt6) function (115). Beyond C3G, the mechanism of Cy in RAFLSs has also been extensively studied. Interleukin-17A (IL-17A) is a crucial pro-inflammatory cytokine in RA, which activates signaling pathways by binding to interleukin-17 receptor subunit A (IL-17RA), driving the expansion of inflammation (119). Samarpita et al. (112) found that Cy effectively interferes with the binding of IL-17A to IL-17RA, thereby significantly inhibiting FLS cell proliferation and migration, and reducing the expression of pathogenic markers such as cysteine-rich angiogenic inducer 61 (cyr61), interleukin-23 (IL-23), granulocyte-macrophage colony-stimulating factor (GM-CSF), and toll-like receptor 3 (TLR3). Additionally, anthocyanin derivatives LRAC and PCA also exhibited inhibitory effects on FLSs. Two studies by Xu et al. (120) and Wu et al. (121) demonstrated that LRAC and PCA significantly inhibited FLS proliferation in a dose-dependent manner. Notably, LRAC did not have a significant negative impact on the growth of T cells and monocytes/macrophages, suggesting that LRAC may possess good targeting and safety profiles (120). PCA, on the other hand, significantly inhibited FLS migration and invasion by reducing the gene expression and secretion levels of TNF-α, IL-1β, and IL-6, and by inhibiting the expression of MMP-3 and MMP-13 (121).

Additionally, the anti-inflammatory effects of Cy, AEBS, and ACNs in RA animal models have been confirmed. Two studies by Samarpita et al. (112, 122) found that in AIA rat models, Cy further significantly reduced the levels of IL-23 and GM-CSF in the serum by inhibiting the phosphorylation of signal transducer and activator of transcription (STAT)3 and the expression of downstream cyr61 and TLR3. This inhibition effectively suppressed inflammatory signal transduction. Moreover, Cy significantly decreased the proportion of T helper 17 (Th17) cells and upregulated the proportion of regulatory T (Treg) cells, thereby restoring immune balance. This effect is achieved by inhibiting the expression of retinoic acid-related orphan receptor gamma t (ROR-γT) and IL-17 while upregulating the expression of forkhead box P3 (FoxP3) and IL-10. Furthermore, Cy also inhibited the expression of T-follicular helper (Tfh) cells and their related factor interleukin-21 (IL-21), while upregulating the proportion of T-follicular regulatory (Tfr) cells, further demonstrating its multifaceted role in regulating adaptive immunity. Another anthocyanin compound, ACNs, has also been validated for its anti-inflammatory effects in AIA rats. He et al. (123) discovered that ACNs significantly reduced the serum levels of TNF-α in AIA rats. AEBS, another representative anthocyanin compound, exhibited significant anti-inflammatory potential in CIA mice models. Min et al. (116) found that AEBS not only significantly decreased the levels of pro-inflammatory cytokines such as IL-1β, IL-6, IL-17, and TNF-α but also effectively inhibited the expression of genes closely related to osteoclast activity, including tartrate-resistant acid phosphatase (TRAP), MMP-9, and RANK. Additionally, multiple studies have further explored the anti-inflammatory effects of Dp GPE, PR, Malβg, and RRE *in vitro* using immune cell lines. These anthocyanin compounds exhibited anti-inflammatory potential by inhibiting the production of pro-inflammatory factors such as IL-6, TNF-α, and IL-1β, regulating the transcription and secretion of inflammatory genes and proteins like MMP-2 and MMP-9, and reducing the activity of key enzymes in inflammatory signaling pathways such as cyclooxygenase-2 (COX-2). Furthermore, they decreased the generation of downstream inflammatory mediators, including prostaglandin E2 (PGE2) and nitric oxide (NO), thereby demonstrating significant anti-inflammatory potential (109–111). In summary, these studies elucidate the anti-inflammatory mechanisms of Cy, DP, GPE, PR, Malβg, C3G, LRAC, AEBS, PCA, RRE, and ACNs in RA from multiple perspectives, providing new scientific evidence for the development of anthocyanin-based therapeutic strategies for RA.

##### 3.2.3.2 The amelioration of joint destruction via oxidative factors

Oxidative stress plays a central role in the onset and persistent development of RA, serving as a crucial mechanism leading to sustained inflammation and joint damage (124). In the inflammatory environment, neutrophils, monocytes, and macrophages in the affected joints of RA patients are highly active, producing large quantities of reactive molecules (125). The most common reactive molecules in the affected joints include superoxide anion, hydrogen peroxide (H₂O₂), hydroxyl radicals, NO, and peroxynitrite (126). The accumulation of these reactive molecules in the local joint environment not only significantly elevates oxidative stress levels but also amplifies the inflammatory response by activating cellular signaling pathways. Multiple studies have demonstrated that AEBS, ACNs, PR, Malβg, and LRAC possess significant potential in alleviating RA-related oxidative stress. Min et al. (116) found that in a CIA mice model, AEBS could inhibit the expression of nitrotyrosine and inducible nitric oxide synthase (iNOS). This indicates that AEBS protects joints and surrounding tissues by reducing the production of reactive molecules through the inhibition of oxidative stress responses. Additionally, He et al. (123) reported that ACNs exhibited significant dual antioxidant and anti-inflammatory effects in an AIA rat model. Specifically, ACNs significantly increased total antioxidant capacity (T-AOC) and SOD activity while decreasing the levels of oxidative damage markers such as MDA (123). This mechanism effectively mitigates RA-related oxidative stress by enhancing the function of the endogenous antioxidant system, thereby reducing inflammation-induced joint damage.

**Figure 5:**
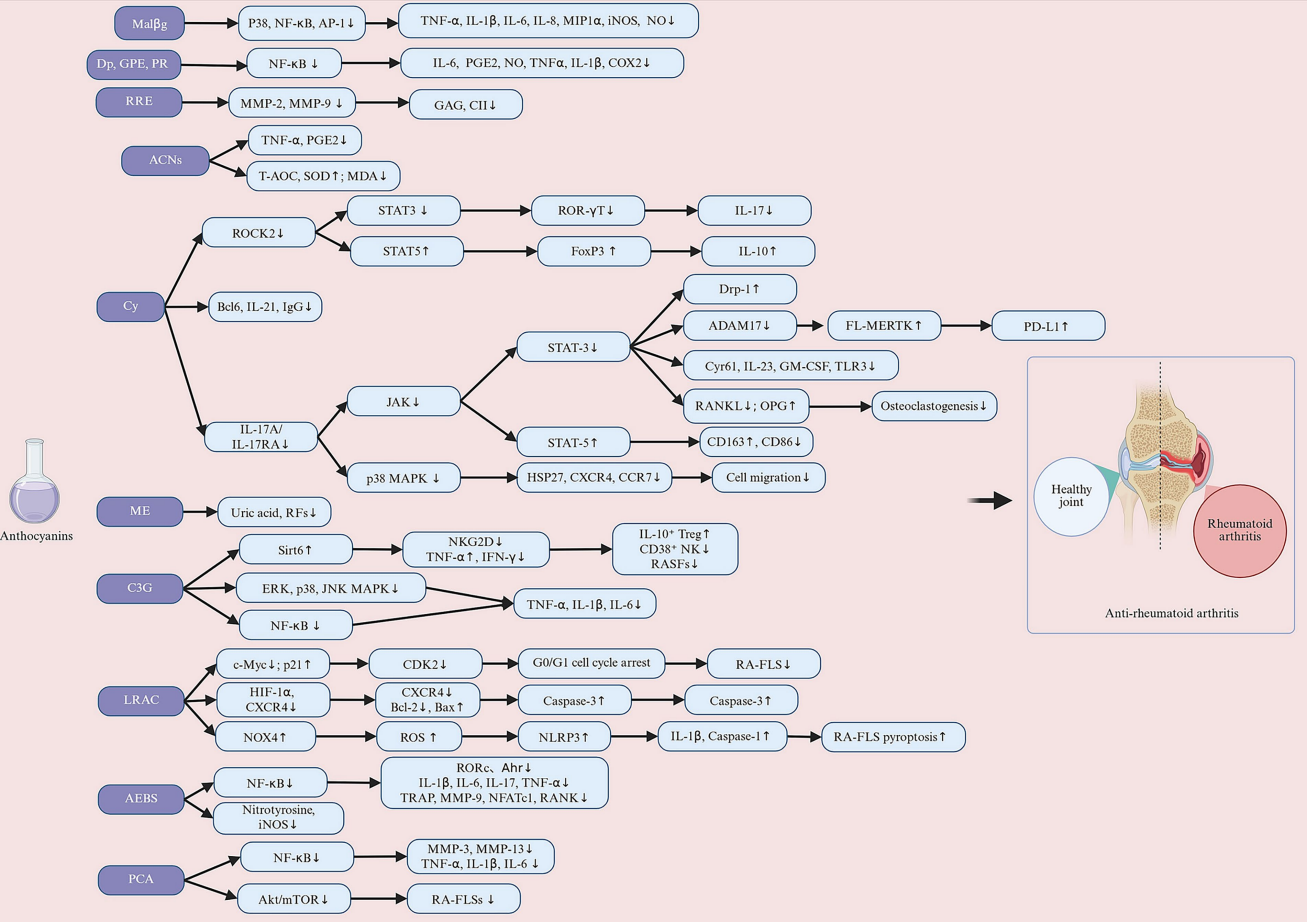
Anthocyanins exert protective effects against rheumatoid arthritis (RA) through multiple molecular mechanisms, including immunomodulation (inhibiting Th17, promoting Treg, regulating Tfh/Tfr, and downregulating CD38^+^ NK cell activity), anti-inflammatory responses (blocking the IL-17A/IL-17RA/JAK-STAT3 pathway, inhibiting NF-κB and MAPKs, reducing FLS proliferation and migration, and suppressing inflammatory factors such as TNF-α, IL-1β, and IL-6), joint protection (inhibiting MMPs/collagenase and osteoclast differentiation), antioxidant/promoting apoptosis body clearance (increasing SOD, T-AOC, inhibiting iNOS, MDA, activating STAT5/MERTK), and epigenetic regulation (inhibiting HATs, reducing NF-κB acetylation, promoting p21/Bax expression to induce cell cycle arrest and apoptosis). Malβg, malvidin-3-O-β glucoside; Dp, delphinidin; GPE, grape polyphenol extract; PR, propolis; RRE, red raspberry extract; ACNs, anthocyanins; Cy, cyanidin; ME, mulberry fruit extract; C3G, cyanidin-3-glucoside; LRAC, *L. ruthenicum* anthocyanins; AEBS, anthocyanin extracted from black soybean seed coats; PCA, protocatechuic acid. Created with BioRender.com.

However, ACNs and their derivatives do not solely aim to reduce the production of reactive molecules in the process of regulating oxidative stress. Xu et al. (120) found that LRAC increased ROS production by upregulating NADPH oxidase 4 (NOX4) expression, but this increase in ROS further activated NLR family pyrin domain containing 3 (NLRP3) inflammatory vesicles and induced IL-1β and interleukin-18 (IL-18) release, which drives focal death. This mechanism suggests that LRAC may attenuate joint inflammation and injury by triggering the programmed cell death pathway and selectively removing hyperproliferative FLS. These findings reveal that AEBS, ACNs, and LRAC modulate oxidative stress processes in RA by enhancing antioxidant defenses and selectively utilizing oxidative stress to induce apoptosis or pyroptosis (Figure 5).

##### 3.2.3.3 The amelioration of joint destruction via NF-κB, MAPK, and JAK/STAT3 signaling pathways

The NF-кB, MAPK, and JAK/STAT3 signaling pathways are classical inflammatory pathways that play a central role in RA and are directly involved in the onset and progression of joint inflammation (127). NF-κB activation is driven by the degradation of IκB proteins and the nuclear translocation of the p65/p50 complex (128). Several studies have now demonstrated that C3G, DP, and PCA effectively intervene in the NF-κB and MAPK signaling pathways through multiple mechanisms, thereby alleviating the inflammatory response in RA. Sun et al. (114) found that C3G significantly reduces the production of pro-inflammatory factors in LPS-induced FLSs by inhibiting the expression of key proteins in the NF-κB and MAPK signaling pathways, such as p65, phosphorylated IκBα, ERK, p38, and JNK (114). Excessive proliferation of FLSs and the release of pro-inflammatory factors are critical drivers of synovial inflammation in RA. Therefore, C3G helps alleviate local joint inflammation by suppressing the NF-κB and MAPK signaling pathways. Seong et al. (129) and Wu et al. (121) found that DP and PCA downregulate the expression of phosphorylated p65 (p-p65) in the NF-κB signaling pathway while stabilizing IκBα, preventing the NF-κB complex from entering the nucleus and thereby inhibiting its transcriptional activity. However, Min et al. (116) found that AEBS did not exhibit significant inhibition of the NF-κB signaling pathway in a CIA mice model, although it showed a trend towards inhibition. Therefore, further research is needed to clarify the beneficial effects of AEBS on the NF-κB signaling pathway. In addition to the NF-κB pathway, the roles of ACNs in the MAPK and JAK/STAT pathways are also noteworthy. Samarpita et al. (112, 122, 130, 131) systematically investigated the regulatory effects of Cy on the MAPK and JAK/STAT pathways in RA, demonstrating its anti-inflammatory effects. Cy significantly inhibits IL-17A-induced monocyte migration and osteoclastogenesis by blocking the p38 and STAT3 pathways, and it reduces inflammation and bone resorption by downregulating IL-17RA expression (131). Additionally, the activation of the JAK/STAT signaling pathway triggered by inflammatory cytokines (IL-6, IL-17, and TNF-α) plays a crucial role in sustaining inflammatory responses and exacerbating disease progression (132). Cy balances T-cell differentiation by inhibiting the rho-associated coiled-coil-containing kinase ROCK-2/STAT3 pathway (122) and regulates IL-17A/IL-17RA-mediated JAK/STAT signaling by blocking the phosphorylation of JAK1/JAK3 and enhancing the expression of protein inhibitor of activated STAT3 (PIAS3) (112). More importantly, Cy further improves the pathological state of RA by restoring the function of impaired synovial macrophages (130). It inhibits the JAK/STAT3 pathway, regulates T-cell differentiation, and restores mitochondrial function and anti-inflammatory macrophage polarization by modulating the expression of a disintegrin and metalloproteinase domain 17 (ADAM17) and dynamin related protein 1 (Drp-1) (130). In summary, C3G, DP, PCA, AEBS, and Cy effectively alleviate inflammatory responses and pathological progression in RA by inhibiting the activation of the NF-κB, MAPK, and JAK signaling pathways and reducing the release of inflammatory factors, demonstrating their great potential as therapeutic strategies. The effects of anthocyanins on rheumatoid arthritis in *in vivo* and *in vitro* studies are summarized in Table 2.

**Table 2:** The effects of anthocyanins on rheumatoid arthritis *in vivo* and *in vitro* studies.

Model		Treatment dose	Treatment route	Signaling pathways/Mechanisms	Intervention outcome	References
*In vivo*/*In vitro*	Animal/Cell					
*In vitro*	IL-1β-induced bovine nasal explant cells	50 μg/mL RRE	N/A	Cartilage matrix catabolism	GAG, CII, MMP-2, MMP-9↓	(111)
*In vivo*	AIA Lewis rats	30, 120 mg/kg/day RRE for 30 days	Gavage			
*In vivo*	AIA Sprague Dawley rats	10–40 mg/kg/day ACNs for 14 days	Oral	Inflammation Oxidative stress	TNF-α, PGE2↓; T-AOC, SOD↑; MDA↓	(123)
*In vitro*	TGF-β, IL-21, IL-6-induced Tfh cells IL-6, TGF-β-induced Th17 cells	10 μM Cy	N/A	ROCK2/STAT	ROR-γT, IgG↓; FoxP3↑; IL-17, IFN-γ, IL-4, IL-21↓; IL-10↑	(122)
*In vivo*	AIA Wistar albino rats	1 mg/kg/day Cy for 10 days	Intraperitoneally			
*In vitro*	IL-17A-induced primary synovial macrophages	10 ng/mL Cy	N/A	JAK/STAT-3	Drp-1, PD-L1, FL-MERTK↑; ADAM17↓	(130)
*In vivo*	AIA Wistar rats	1 mg/kg Cy for 10 days	Intraperitoneally			
*In vitro*	TNF-α-induced MH7A cells LPS-induced Jurkat cells	10, 30 μM DP	N/A	NF-κB Inflammation	IL-6, COX-2, IL-1β, TNF-α↓	(129)
*In vitro*	CD23-induced MDM LPS-induced macrophages	50 μM Malβg	N/A	Inflammation	TNF-α, IL-1β, MIP1α, IL-8, IL-6, iNOS, Nitrites, NO↓	(109)
*In vivo*	AIA Lewis rats	25 mg/kg/day Malβg for 5 days	Oral			
*In vitro*	LPS-induced PBLs	12.5 μg/mL GPE; 25 μg/mL PR	N/A	Inflammation	TNF-α, IL-1β, IL-6, IL-8, IL-10, PGE2, NO, IFN-γ↓	(110)
*In vivo*	AIA Lewis rats	50, 250 mg/kg GPE, PR for 50 days	Oral			
*In vitro*	RA-FLS; MNCs CD38^+^ NK cells	0–100 μM C3G	N/A	Inflammation Cell apoptosis	IL-2, IL-10, Sirt6, TNF-α↑; IFN-γ, IL-6, NKG2D↓	(115)
*In vivo*	CIA Sprague Dawley rats	25 mg/kg C3G for 3 weeks	Injection			
*In vitro*	IL-17-induced FLS	5–10 μM Cy	N/A	JAK/STAT-3 Inflammation	Cyr61, IL-23, GM-CSF, TLR3, IL-17RA, p-STAT-3↓	(112)
*In vivo*	AIA Wistar rats	1 mg/kg/day Cy for 11 days	intraperitoneally			
*In vivo*	Carrageenan-induced arthritic rat model	50 mg/kg ME	Oral	Inflammation	Uric acid, RFs↓	(113)
*In vitro*	LPS-induced FLS	5-40 μM C3G	N/A	NF-κB; ERK, p38, JNK Inflammation	TNF-α, IL-1β, IL-6↓	(114)
*In vivo*	CIA B6 mice	50 mg/kg/day C3G for 7 days	Intraperitoneally			
*In vitro*	RA-FLS cells	400 μg/mL LRAC	N/A	Cell apoptosis Oxidative stress	ROS, NOX4, NLRP3, IL-1β, caspase-1↑; HIF-1α, CXCR4↓	(120)
*In vitro*	CD4^+^ T cells M-CSF, RANKL-induced BMMs, PBMCs	50–200 μg/mL AEBS	N/A	NF-κB Inflammation Oxidative stress	TRAP, MMP-9, NFATc1, RANK, IL-1β, IL-6, IL-17, TNF-α↓; Nitrotyrosine, iNOS↓	(116)
*In vivo*	CIA DBA/1J mice	60 mg/kg/day AEBS for 7 weeks	N/A			
*In vitro*	IL-17-induced monocyte, FLS	7.5, 10 μM Cy	N/A	p38 IL-17A/STAT-3	HSP27, CXCR4, CCR7↓; RANKL, IL-17RA↓, OPG↑	(131)
*In vitro*	RA-FLS cells	5-20 μM PCA	N/A	NF-κB Inflammation Cell apoptosis	MMP-3, MMP-13, TNF-α, IL-1β, IL-6↓	(121)

↑, increased; ↓, decreased. RRE, red raspberry extract; GAG, glycosaminoglycan; CII, type II collagen; AIA, adjuvant-induced arthritis; ACNs, anthocyanins; PGE2, prostaglandin E2; T-AOC, total antioxidant capacity; SOD, superoxide dismutase; STAT, signal transducer and activator of transcription; ROCK, rho-associated coiled-coil-containing kinase; C3G, cyanidin-3-glucoside; IL-1β, interleukin-1 beta; IL-6, interleukin-6; TGF-β, transforming growth factor-beta; ROR-γT, retinoic acid-related orphan receptor gamma t; IgG, immunoglobulin G; FoxP3, forkhead box P3; IL-17, interleukin-17; IFN-γ, interferon-gamma; IL-4, interleukin-4; IL-21, interleukin-21; IL-10, interleukin-10; IL-17A, interleukin-17A; Drp-1, dynamin-related protein 1; PD-L1, programmed death ligand-1; ADAM17, a disintegrin and metalloproteinase domain 17; DP, delphinidin; Cy, cyanidin; COX-2, cyclooxygenase-2; MDM, monocytes-derived macrophages; Malβg, malvidin-3-O-β glucoside; MIP1α, macrophage inflammatory protein 1alpha; IL-8, interleukin-8; iNOS, inducible nitric oxide synthase; NO, nitric oxide; PBLs, peripheral blood-derived mononuclear leukocytes; GPE, grape polyphenol extract; PR, propolis; RA, rheumatoid arthritis; FLS, fibroblast-like synoviocytes; MNCs, mononuclear cells; IL-2, interleukin-2; Sirt6, sirtuin 6; NKG2D, natural killer group 2D; CIA, collagen-induced arthritis; Cyr61, cysteine-rich angiogenic inducer 61; IL-23, interleukin-23; GM-CSF, granulocyte-macrophage colony-stimulating factor; TLR3, toll-like receptor 3; IL-17RA, interleukin-17 receptor subunit A; ME, mulberry fruit extract; RFs, rheumatoid factors; LRAC, *L. ruthenicum* anthocyanins; ROS, reactive oxygen species; NOX4, NADPH oxidase 4; NLRP3, NLR family pyrin domain containing 3; HIF-1α, hypoxia-inducible factor 1 alpha; CXCR4, C-X-C chemokine receptor type 4; PBMCs, peripheral blood mononuclear cells; AEBS, anthocyanin extracted from black soybean seed coats; TRAP, tartrate-resistant acid phosphatase; RANK, receptor activator of nuclear factor-kappa B; HSP27, heat shock protein 27; CCR7, CC-chemokine receptor 7.

### 3.3 Anthocyanins and osteoarthritis

#### 3.3.1 Overview of osteoarthritis

OA, also known as osteoarthrosis, is the most common progressive chronic joint disease among the elderly, leading to significant chronic pain, reduced mobility, and disability (133). Currently, studies on inflammatory factor- and oxidative stress-induced chondrocyte inflammation models, as well as the destabilization of the medial meniscus (DMM) mouse model and monosodium iodoacetate (MIA) rat model, have provided an important foundation for exploring the pathogenesis of OA and potential therapeutic strategies. Therefore, this review aims to summarize current information on the potential efficacy of ACNs in OA treatment and to analyze and discuss their mechanisms of action and research findings.

#### 3.3.2 Effects of anthocyanins on osteoarthritis animal studies

The pathological features of OA include degeneration of cartilage, loss of proteoglycans, joint pain, and dysfunction, which are usually accompanied by an amplified inflammatory response, increased tissue damage, and abnormally enhanced pain perception (134). Researchers explored the *in vivo* protective effects of Cy and Pg by establishing an OA model in mice through DMM surgery. The DMM model is a commonly used mechanical injury model that simulates the chronic progression of cartilage degeneration and inflammatory response in human OA. Studies have shown that Cy attenuates the development of OA by reducing proteoglycan loss and cartilage destruction (5). Zeng et al. (135) found that Pg concentration-dependent improvement of articular cartilage in DMM-induced OA mice. This was specifically demonstrated by the restoration of cartilage surface morphology and cartilage thickness to near normal levels. Thus, ACNs are not only capable of delaying the disease course by reducing cartilage degeneration and matrix degradation, but may also promote chondrocyte repair and regenerative capacity in addition to chondroprotection and tissue repair. The role of ACNs in pain modulation has also attracted much attention. The MIA model, a chemically induced model of OA, mimics the onset of acute joint pain by destroying chondrocytes and inducing inflammation (136). Dai et al. (137) investigated the analgesic effect of Mv through the MIA-induced OA rat model. The findings showed that Mv was enough to significantly increase the nociceptive threshold of MIA rats, while significantly improving the limb withdrawal threshold (LWT) by continuous treatment, suggesting its reversal effect on joint pain (137). These findings reveal multiple mechanisms of Cy, Mv and PG in OA treatment, including cartilage protection, tissue repair, and pain modulation.

#### 3.3.3 Molecular mechanisms of anthocyanins in the prevention of osteoarthritis

##### 3.3.3.1 The amelioration of joint destruction via inflammatory factors

Chondrocytes are the only cells present in cartilage with the function of synthesizing extracellular matrix (ECM) and maintaining cartilage homeostasis, and an imbalance in ECM is thought to be the direct cause of OA (138). Numerous studies have demonstrated that C3G, peonidin-3-O-glucoside (P3G), CC, Peonidin chloride (PC), PCA, Cy, Mv, Pg, and Dp effectively inhibit ECM degradation, reduce chondrocyte apoptosis, and play an important role in maintaining cartilage homeostasis (5, 135, 137, 139, 140). The pathogenesis of OA is closely related to chronic inflammation, and the role of inflammatory factors in chondrocyte metabolism is particularly significant (141). Pro-inflammatory factors such as IL-1β, IL-6, and TNF-α are key mediators in the development of OA. They promote the synthesis of matrix-degrading enzymes, such as MMPs and ADAMTS, by activating a series of signaling pathways (142). Additionally, inflammatory mediators such as NO, PGE2, and COX-2 also play important roles in cartilage synthesis and degradation. Increased NO levels inhibit the production of proteoglycans and CII, leading to oxidative damage and chondrocyte death (143, 144), while elevated PGE2 reduces ECM synthesis, inhibits chondrocyte proliferation, and promotes cartilage degradation (145). Current research has demonstrated that Cy, Mv, Dp, and Pg significantly inhibit pro-inflammatory cytokines. Jiang et al. (5) found that Cy dose-dependently inhibits the production of inflammatory mediators, including iNOS, COX-2, PGE2, NO, TNF-α, and IL-6, in IL-1β-induced human OA chondrocytes (5). Similarly, Zeng et al. (135) and Dai et al. (137) showed that Pg and Mv suppress the expression of inflammatory factors such as TNF-α, IL-6, IL-1β, COX-2, and iNOS (137), further highlighting the anti-inflammatory potential of ACNs. Moreover, ACNs alleviate inflammatory responses in OA chondrocytes by regulating the nuclear factor kappa-B (NF-κB) signaling pathway. Haseeb et al. (146) found that Dp significantly reduces COX-2 expression and PGE2 production in IL-1β-induced OA chondrocytes by inhibiting the NF-κB signaling pathway. Additionally, inflammatory factors significantly increase the mRNA and protein expression levels of MMPs in chondrocytes (142), and anthocyanin compounds dose-dependently inhibit the expression of these MMPs. Multiple studies have shown that Mv, Pg, Cy, C3G, pelargonidin-3-O-glucoside (Pg-3-glc), P3G, PCA, C3G, P3G, CC and PC effectively inhibit the expression and secretion of MMP-1, MMP-3, and MMP-13 in chondrocytes induced by IL-1β and advanced glycation end products (AGEs) (5, 135, 137, 139, 147). In addition to their inhibitory effects on MMPs, Cy and Pg dose-dependently increase the synthesis of CII and aggrecan while suppressing a disintegrin and metalloproteinase with thrombospondin motifs 5 (ADAMTS5) expression, further supporting their potential in protecting articular cartilage and maintaining ECM homeostasis (5, 135). In summary, Cy, Mv, Dp, Pg, C3G, Pg-3-glc, P3G, PCA, C3G, P3G, CC and PC not only inhibit the production of pro-inflammatory cytokines and matrix-degrading enzymes but also promote the synthesis of cartilage matrix, preserving the structure and function of cartilage.

##### 3.3.3.2 The amelioration of joint destruction via oxidative factors

The pathogenesis of OA is complex and multifaceted, with oxidative stress considered a central component. As a core mechanism in OA pathogenesis, oxidative stress triggers chondrocyte apoptosis and synovial inflammation through the accumulation of ROS, leading to the degeneration and destruction of joint tissues (144). In this process, Dp has demonstrated significant protective effects against oxidative stress. Lee et al. (140) found that Dp effectively protects human chondrocytes from oxidative stress damage through its antioxidant properties and autophagy-activating effects. Specifically, Dp treatment inhibits the increase in ROS levels induced by H₂O₂ and reduces oxidative damage. Additionally, Dp exerts protective effects by regulating key signaling pathways. The activation of nuclear factor (erythroid-derived 2)-like 2 (Nrf2) promotes the synthesis of antioxidant enzymes, thereby enhancing the cell’s ability to scavenge ROS (148). Oxidative stress leads to antioxidant imbalance, but Dp can activate the NF-κB pathways, maintaining redox balance (140). Notably, oxidative stress not only induces apoptosis but also disrupts the dynamic balance of intracellular autophagy, which is considered a crucial mechanism for maintaining cellular homeostasis (149). Studies have shown that Dp effectively enhances cellular autophagy by increasing the levels of the autophagy-related protein LC3-II and promoting autophagosome formation, thereby clearing waste and damaged organelles accumulated due to oxidative damage (140). At the same time, Dp significantly regulates apoptotic signaling. Dp treatment markedly increases the expression of the anti-apoptotic protein B-cell lymphoma-extra large (Bcl-xl) and reduces the activity of pro-apoptotic proteins caspase-3 and cleaved poly (ADP-ribose) polymerase (c-PARP). This effect not only prevents chondrocyte apoptosis but also protects the integrity of the cartilage matrix by mitigating ROS accumulation (140). In summary, oxidative stress plays a critical role in the pathogenesis of OA, and Dp demonstrates significant protective effects against oxidative stress through multiple mechanisms, including inhibiting ROS elevation, activating the Nrf2 and NF-κB signaling pathways, inducing autophagy, and regulating apoptosis-related proteins.

##### 3.3.3.3 The amelioration of joint destruction via NF-κB and MAPK signaling pathways

In the pathogenesis of OA, multiple signaling pathways and inflammatory responses are involved, among which the NF-κB signaling pathway serves as a central regulator of inflammation. NF-κB signaling pathway is primarily divided into the canonical and non-canonical pathways. In the canonical pathway, NF-κB exists as a p65/p50 heterodimer bound to the IκB, maintaining it in an inactive state within the cytoplasm (150). Upon stimulation by inflammatory cytokines, IκB is rapidly phosphorylated by the IκB kinase (IKK) complex, allowing NF-κB to become activated and translocate to the nucleus, thereby promoting the transcription of MMPs genes, ultimately exacerbating cartilage degradation and the inflammatory response (151). Recent studies have demonstrated that ACNs and their metabolites play significant roles in inhibiting the NF-κB signaling pathway. Wongwichai et al. (139) thoroughly investigated how ACNs (C3G, P3G) and their metabolites (CC, PC, and PCA) can block the activation of the IL-1β-induced NF-κB pathway by inhibiting IKK activity and the phosphorylation of IκBα. Notably, the deglycosylated metabolites (CC, PC, PCA) exhibited stronger inhibitory effects (139). This indicates that the deglycosylated forms are more effective than their glycosylated counterparts in suppressing the NF-κB signaling. Similarly, Chuntakaruk et al. (147) found that C3G, Pg-3-glc, P3G, and PCA dose-dependently inhibited the phosphorylation of IKK, IκB, and p65 in the AGEs-mediated NF-κB signaling pathway. This suggests that ACNs can not only alleviate IL-1β-induced inflammation but also suppress AGEs-mediated pro-inflammatory responses. Additionally, another study discovered that PG inhibits the activation of the NF-κB pathway by suppressing the nuclear translocation of p65 in chondrocytes, thereby further mitigating the inflammatory response (135). Haseeb et al. (146) found that Dp inhibits the activation of the NF-κB signaling pathway by preventing the degradation of IκBα, downregulating the activity and expression of the IKKα and IKKβ complexes, and blocking the activation of NF-κB-inducing kinase (NIK) and IL-1 receptor-associated kinase-1 (IRAK1). Moreover, Sirt6 directly interacts with NF-κB (p65), mediating the deacetylation of histone H3, thereby inhibiting the transcription of NF-κB target genes (152). Jiang et al. (5) discovered that Cy inhibits the NF-κB pathway by activating Sirt6, which suppresses the phosphorylation of NF-κB p65 and IκBα induced by IL-1β. Notably, Mv regulates the NF-κB signaling pathway through a mechanism distinct from classical inhibitors. Dai et al. (137) found that although Mv can inhibit the NF-κB pathway by blocking the nuclear translocation of p65, this process does not rely on the degradation of IκBα or the activation of IKKβ. Further research revealed that Mv’s effect may primarily occur through the non-canonical NF-κB pathway. In the non-canonical pathway, activated NIK triggers the phosphorylation of IKKα, leading to the degradation of the NF-κB2 precursor (p100) and the release of NF-κB2 (p52), which forms a heterodimer with RelB to enter the nucleus and regulate the expression of specific genes (153). Mv may selectively inhibit the non-canonical pathway by interfering with NIK activity or p100 degradation, thereby exerting its effects independently of IκBα. In summary, C3G, P3G, CC, PC, Pg-3-glc, PCA, Dp, and PG primarily exert their anti-inflammatory effects by regulating the canonical NF-κB signaling pathway, while Cy provides a novel approach through epigenetic regulation by activating Sirt6. In contrast, Mv demonstrates a unique mechanism by intervening in the non-canonical NF-κB pathway.

**Figure 6:**
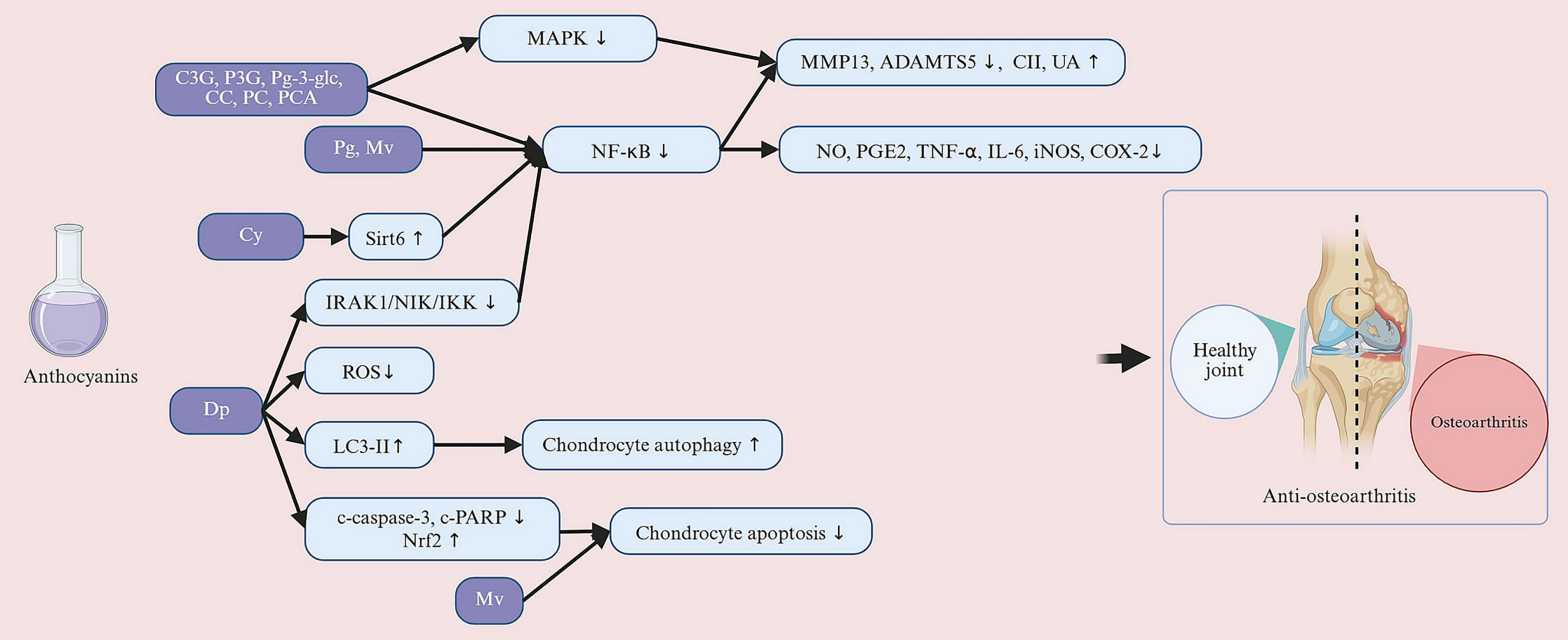
Anthocyanins exert their protective effects against osteoarthritis through a multi-target mechanism. Their anti-inflammatory effects include inhibiting the IRAK-1/NIK/IKK pathway, blocking IκBα degradation, activating Sirt6, downregulating NF-κB signaling, and inhibiting MAPK pathway phosphorylation; simultaneously, they induce autophagy and activate the Nrf2 pathway to reduce oxidative stress and cell apoptosis. These mechanisms collectively suppress the expression of inflammatory mediators (such as MMPs and ADAMTS5), promote the synthesis of matrix components such as collagen II, aggrecan, and GAGs, thereby inhibiting cartilage degradation, exerting a cartilage-protective effect, and delaying the progression of joint structural damage. C3G, cyanidin-3-glucoside; P3G, peonidin-3-O-glucoside; Pg-3-glc, pelargonidin-3-O-glucoside; CC, cyanidin chloride; PC, peonidin chloride; PCA, protocatechuic acid; Pg, pelargonidin; Mv, malvidin; Cy, cyanidin; Dp, delphinidin. Created with BioRender.com.

In the pathogenesis of OA, the MAPK signaling pathway plays a crucial role. Specifically, the MAPK family includes ERK, p38 kinase, and JNK. These kinases are activated through phosphorylation and regulate processes such as inflammatory responses and ECM degradation (154). Studies have shown that the phosphorylation levels of key molecules in the MAPK signaling pathway (e.g., p-ERK, p-p38, and p-JNK) are significantly elevated in the cartilage of OA patients. These alterations are closely associated with the release of inflammatory cytokines and the degradation of the cartilage matrix (155, 156). Wongwichai et al. (139) further investigated the regulatory effects of ACNs (such as C3G and P3G) and their metabolites (CC, PC, and PCA) on the MAPK signaling pathway. ERK phosphorylation was significantly inhibited by C3G, P3G, CC, PC, and PCA under IL-1β stimulation, and especially the deglycosylated forms (CC, PC, and PCA) showed stronger inhibitory effects. Also, the study revealed differential roles of ACNs and their metabolites in the p38 and JNK signaling pathways. p38 phosphorylation was inhibited only with P3G, JNK phosphorylation was significantly inhibited only with PC treatment, and PCA showed a complex mechanism of action that both enhanced JNK phosphorylation and exhibited a superimposed effect when co-treated with IL-1β (139). Similarly, Chuntakaruk et al. (147) found that C3G and P3G significantly decreased the phosphorylation levels of ERK, p38, and JNK, in contrast to Pg-3-glc, which also significantly inhibited the activation of ERK and JNK, but had no effect on the phosphorylation level of p38. These studies suggest that C3G, P3G, CC, PC, PCA, and Pg-3-glc regulate the MAPK signaling pathway through different mechanisms (Figure 6). The role of different types of ACNs and their metabolites in the MAPK signaling pathway is somewhat specific, and the specificity of the regulation of the MAPK family by different ACNs needs to be explored in depth in the future. The effects of anthocyanins on osteoarthritis in *in vivo* and *in vitro* studies are summarized in Table 3.

**Table 3:** The effects of anthocyanins on osteoarthritis *in vivo* and *in vitro* studies.

Model		Treatment dose	Treatment route	Signaling pathway/Mechanisms	Intervention outcome	References
*In vivo*/*In vitro*	Animal/Cell					
*In vitro*	IL-1β-induced HAC OSM, IL-1β-induced porcine cartilage degradation	2.5–10 μM C3G, P3G, CC, PC, PCA 6.25–50 μg/mL C3G, P3G, PCA	N/A	NF-κB; ERK Degradation of extracellular matrix Inflammation	MMP-1, MMP-3, MMP-13, s-GAG, HA, hydroxyproline↓; CII, UA↑	(139)
*In vitro*	AGEs-induced porcine cartilage degradation AGEs-induced HAC	6.25-25 μg/mL C3G, Pg-3-glc, P3G, PCA 2.5-10 μM Pg-3-glc, P3G, PCA, C3G	N/A	NF-κB; ERK, p38, JNK Inflammation	s-GAG, HA, MMP-1, MMP-3, MMP-13↓; UA↑	(147)
*In vitro*	IL-1β-induced human OA chondrocytes	12.5 μM, 25 μM, 50 μM Cy	N/A	NF-κB Degradation of extracellular matrix Inflammation	iNOS, COX-2, PGE2, NO, TNF-α, IL-6, MMP13, ADAMTS5↓; CII, Sirt6, Aggrecan↑	(5)
*In vivo*	DMM-induced OA C57BL/6 mice	50 mg/kg/day Cy for 8 weeks	Gavage			
*In vitro*	IL-1β, TNF-α-induced chondrocytes	10, 50 μg/mL Dp	N/A	NF-κB Inflammation	COX-2, PGE2↓	(146)
*In vivo*	MIA-induced OA Wistar rats	10, 20 mg/kg/day Mv for 14 days	Gavage	NF-κB Chondrocyte apoptosis Inflammation Cartilage matrix catabolism	TNF-α, IL-6, IL-1β, MMP-3, MMP-9, MMP-13↓	(137)
*In vitro*	IL-1β-induced chondrocytes	0–40 μM Pg	N/A	NF-κB Inflammation Degradation of extracellular matrix Cartilage damage Cartilage matrix catabolism	IL-6, TNF-α, COX-2, iNOS, ADAMTS5, MMP13↓; Aggrecan, CII ↑	(135)
*In vivo*	DMM -induced OA C57BL/6J mice	10, 20 mg/kg/day Pg for 8 weeks	Oral			
*In vitro*	H_2_O_2_-induced C28/I2 human chondrocyte	40 μM Dp	N/A	NF-κB Chondrocyte apoptosis Chondrocyte autophagy Oxidative stress	ROS↓; Nrf2↑; c-caspase-3, c-PARP↓; Bcl-xl, LC3-II↑	(140)

↑, increased; ↓, decreased. HAC, human articular chondrocytes; OSM, oncostatin M; P3G, peonidin-3-O-glucoside; PC, peonidin chloride; s-GAG, sulfated glycosaminoglycan; HA, hyaluronic acid; UA, uronic acid; AGEs, advanced glycation end products; Pg-3-glc, pelargonidin-3-O-glucoside; OA, osteoarthritis; iNOS, inducible nitric oxide synthase; PGE2, prostaglandin E2; ADAMTS5, a disintegrin and metalloproteinase with thrombospondin motifs 5; DMM, destabilization of the medial meniscus; MIA, monosodium iodoacetate; Mv, malvidin; Pg, pelargonidin; Nrf2, nuclear factor (erythroid-derived 2)-like 2; c-PARP, cleaved poly (ADP-ribose) polymerase; Bcl-xl, B-cell lymphoma-extra large.

### 3.4 Anthocyanins and osteosarcoma

OS results from abnormal differentiation and malignant proliferation of osteoblasts, most of which are located in the epiphyses of long bones, such as the distal femur and proximal tibia (157). The development and progression of OS is associated with aberrant activation of multiple cell signaling pathways, including Wnt/β-catenin, PI3K/AKT, MAPK signaling pathway (158). Studies have shown that ACNs exhibit regulatory effects on multiple signaling pathways, inhibiting the growth and migration of OS cells through various mechanisms, while simultaneously inducing apoptosis and autophagy. Apoptosis, as a form of programmed cell death, is crucial for maintaining cellular homeostasis and preventing inflammatory responses. Specifically, Pg, ACNs and C3G all demonstrate a concentration-dependent induction of G2/M phase cell cycle arrest in OS cells, thereby inhibiting their proliferation and inducing autophagic apoptosis (159–163). Although Dp induces autophagy markers (LC3-II, autophagosome formation), this autophagy has a cell-protective effect. Combining autophagy inhibitors (3-MA, bleomycin A1) can synergistically enhance the anticancer effect (162). This suggests that the autophagy induced by Dp has a cell-protective effect on osteosarcoma cells rather than a mechanism leading to cell death. Therefore, inhibiting protective autophagy is key to enhancing efficacy, and the combination therapy strategy of Dp with autophagy inhibitors should be considered. Further mechanistic studies indicate that ACNs can induce cell death by promoting the accumulation of ROS within cells. In U2OS cells, apoptotic cell death was triggered through ROS-related pathways, rather than autophagic cell death (162). Additionally, Pg acts through a similar mechanism. It not only increases ROS generation and promotes the decrease of mitochondrial membrane potential, but further upregulates the expression of autophagy-related proteins LC3-II and Beclin-1, which ultimately leads to autophagic apoptosis of cells (163). Excessive accumulation of ROS may cause irreversible damage to tumor cells by disrupting mitochondrial function, triggering oxidative stress, and causing signal signaling pathway dysregulation, thus playing a crucial role in the antitumor effects of ACNs.

In addition, the PI3K/AKT signaling pathway is one of the core regulatory signaling pathways for cancer cell growth, survival and migration, and aberrant activation of this is an important mechanism for OS development (164). Pg plays a key role in OS cells by inhibiting the PI3K/AKT signaling pathway (163). Specifically, Pg was able to significantly reduce the levels of p-PI3K and p-AKT protein expression, thereby inhibiting the activity of this signaling pathway (163). This process was accompanied by the expression of autophagy-related proteins LC3-II and Beclin-1, suggesting that Pg is able to induce autophagic death in OS cells through the inhibition of the PI3K/AKT signaling pathway (163). Further emphasizes the critical role of autophagy in Pg-mediated cell death. The role of Dp in OS cells was further supported by the study of Kang et al. (160). It was found that Dp was able to reduce cell viability with inhibition of OS cell proliferation, mainly through induction of mitochondria-dependent apoptosis (160). Specifically, Dp activated caspase-3 and PARP by down-regulating Bcl-2 and up-regulating Bak, and triggered the release of cytochrome c from mitochondria, ultimately leading to apoptosis. Dp also time-dependently reduced the phosphorylation levels of ERK1/2 and p38 in HOS and U2OS cells, thereby blocking the epithelial-to-mesenchymal transition. This suggests that Dp inhibits migration of OS cells mainly by inhibiting ERK1/2 and p38 phosphorylation in the MAPK signaling pathway.

**Figure 7:**
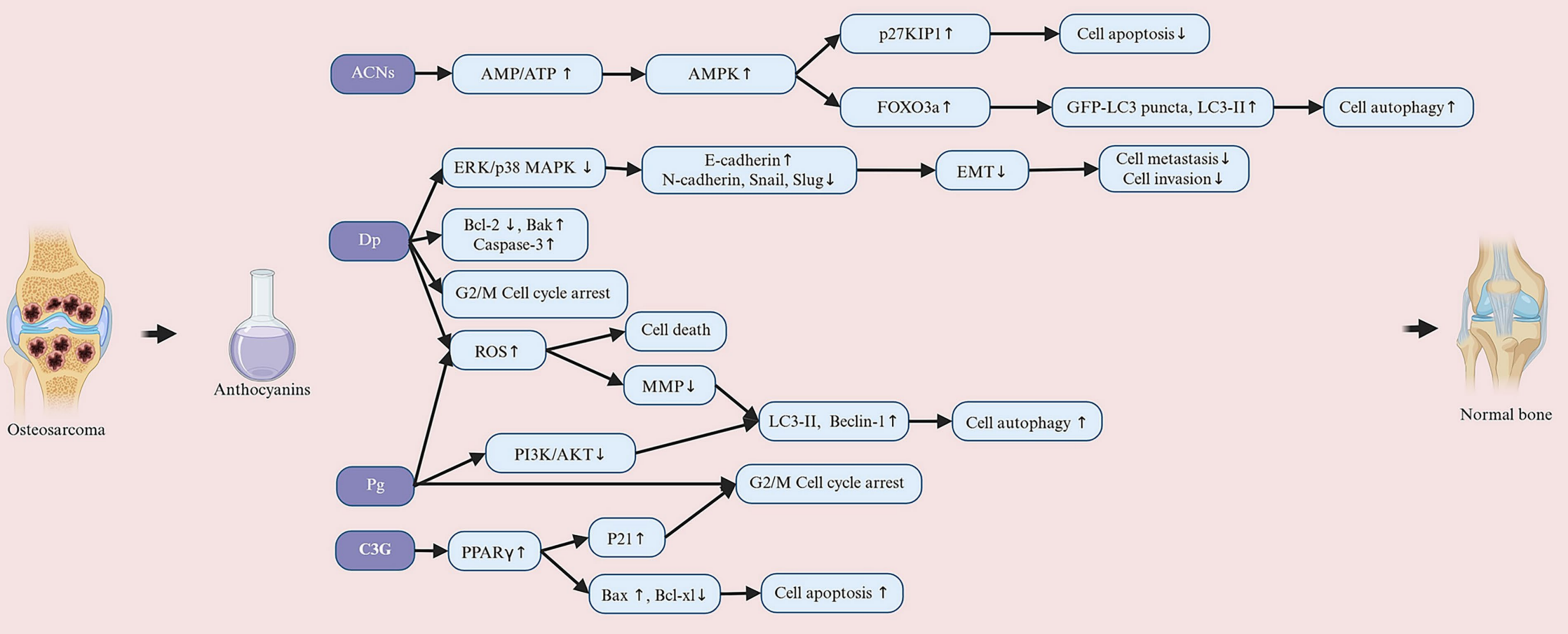
Anthocyanins regulate the survival and metastasis of osteosarcoma cells through multiple signaling pathways. The mechanisms include activating the AMPK pathway, inducing reactive oxygen species (ROS) production, and reducing mitochondrial membrane potential, thereby promoting autophagy by inhibiting mTOR, leading to autophagic cell death; inhibiting the PI3K/AKT signaling pathway, promoting the apoptosis pathway, regulating the expression of Bax/Bak, Bcl-2, and caspase-3, and inducing cell apoptosis; upregulating PPARγ and cell cycle regulatory factors P21/P27 to induce G2/M phase cell cycle arrest and inhibit cell proliferation; and inhibiting the MAPK/ERK/p38 signaling pathway to suppress epithelial-mesenchymal transition (EMT), increase E-cadherin expression, reduce N-cadherin, snail, and slug expression, and decrease metastatic potential. ACNs, anthocyanins; Dp, delphinidin; Pg, pelargonidin; C3G, cyanidin 3-O-b-d-glucoside. Created with BioRender.com.

Moreover, the anti-tumor effect of C3G in OS cells was also achieved mainly by inducing apoptosis rather than necrosis, and the effect varied depending on the OS cell type (159). Specifically, after C3G treatment, peroxisome proliferator-activated receptor gamma (PPARγ), Bcl-2-associated X protein (Bax), and cyclin-dependent kinase inhibitor 1 (P21) were significantly increased, with a more pronounced increase in P21 expression in MG-63 cells and a decrease in Bcl-xl expression (159). In addition to the PI3K/AKT and MAPK signaling pathways, the adenosyl monophosphate-dependent protein kinase (AMPK) signaling pathway also plays an important role in anthocyanin-mediated antitumor effects. Choe et al. (161) found that ACNs were able to, through activation of AMPK signaling pathway inducing autophagy in U2OS cells. Specifically, ACNs promote autophagy through AMPK-mediated activation of Forkhead box O3 (FOXO3a) and simultaneously inhibit apoptosis by upregulating the expression of p27KIp1 (161). This dual regulatory role of AMPK not only facilitates the occurrence of autophagy but also appropriately limits apoptosis. Moreover, ACNs exhibit significant selective toxicity, demonstrating specific cytotoxic effects on cancer cells while exhibiting low toxicity towards normal cells. Extracts of ACNs from Begonia malabarica and Begonia rex-cultorum “Baby Rainbow” showed toxicity towards malignant tumor cells (HT29, HeLa, MG63), resulting in reduced cell numbers and cell rounding, whereas they exhibited no toxic effects on fibroblasts (L929) (165). This selective cytotoxicity suggests that ACNs not only hold potential for cancer therapy but may also reduce the common side effects associated with traditional anticancer drugs. Collectively, these studies indicate that Pg, Dp, ACNs, C3G, and anthocyanin extracts from Begonia malabarica and Begonia rex-cultorum “Baby Rainbow” can significantly reduce the viability of cancer cells by regulating autophagy and apoptosis pathways. By promoting ROS generation, inhibiting the PI3K/AKT signaling pathway, activating AMPK, and modulating MAPK, these compounds not only enhance the expression of autophagy-related proteins but also suppress cell proliferation and migration, demonstrating selective cytotoxic potential against cancer cells and highlighting their promise in cancer treatment (Figure 7). The effects of anthocyanins on osteosarcoma in *in vivo* and *in vitro* studies are summarized in Table 4.

**Table 4:** Subgroup analysis of curcumin treatment on bone mineral density and trabecular microstructure.

Model		Treatment dose	Treatment route	Signaling pathways/Mechanisms	Intervention outcome	References
*In vivo*/*In vitro*	Animal/Cell					
*In vitro*	U2OS cells	0–30 μM Pg	N/A	PI3K/AKT Cells autophagy Cell cycle arrest	LC3-II, Beclin-1↑; LC3-I↓; ROS↑	(163)
*In vitro*	HOS, MG-63, U2OS cells	75 μM Dp	N/A	ERK, p38 Cell apoptosis Cell metastasis Cell invasion	Bak, caspase-3, PARP↑; Bcl-2↓; E-cadherin↑; N-cadherin, Snail, Slug↓	(160)
*In vitro*	U2OS cells	0–200 μM Dp	N/A	Cell apoptosis Cell cycle arrest Cell autophagy Cell death	P62↓; LC3-II↑, ROS↑	(162)
*In vitro*	U2OS cells	0–300 μg/mL ACNs	N/A	AMPK Cell autophagy Cell apoptosis	p27KIP1, FOXO3a ↑ GFP-LC3 puncta, LC3-II↑	(161)
*In vitro*	Saso-2, MG-63, G-292 cells	110–140 μg/mL C3G	N/A	Cell apoptosis Cell cycle arrest	PPARγ, P21↑ Bax↑, Bcl-xl↓	(159)

↑, increased; ↓, decreased. Pg, pelargonidin; PARP, poly (ADP-ribose) polymerase; AMPK, adenosyl monophosphate-dependent protein kinase; PPARγ, peroxisome proliferator-activated receptor gamma; Bax, Bcl-2-associated X protein.

## 4 Discussion

ACNs are a class of naturally occurring water-soluble pigments found in plants that have shown great potential in improving MSDs such as OP, RA, OA, and OS. ACNs positively impact MSDs through a variety of mechanisms, including antioxidant, anti-inflammatory, immunomodulatory, and modulation of bone metabolic signaling pathways. These mechanisms not only reduce disease-induced inflammation and tissue damage, but also promote bone formation and cartilage repair. In the study of OP, ACNs promote bone formation by up-regulating osteogenesis-related genes (e.g., Runx2, ALP, and Ocn) and their signaling pathways (e.g., Wnt/β-catenin), and at the same time, they can restore the balance of bone metabolism by modulating the ratio of RANKL/OPG, inhibiting the NF-κB and MAPK signaling pathways, inhibiting the generation of ROS, and reducing the pro-inflammatory factors and thus inhibiting the activity of osteoclasts. Metabolic homeostasis. In RA, ACNs reduce the release of inflammatory factors by inhibiting the activation of NF-кB, MAPK, and JAK/STAT3 signaling pathways and restore the balance of the immune system by inhibiting oxidative stress. In OA, ACNs effectively reduce chondrocyte apoptosis and extracellular matrix degradation, and also inhibit the expression of inflammatory factors by inhibiting NF-κB and MAPK signaling pathways. In OP, RA, and OA, ACNs attenuate the damage of oxidative stress on bone and chondrocytes by reducing the accumulation of ROS. In contrast, in OS, exhibits anticancer effects. It inhibits cancer cell proliferation and migration by promoting ROS generation, inhibiting the PI3K/AKT signaling pathway, activating AMPK and regulating MAPK, and induces autophagic death of cancer cells by promoting autophagy-related proteins such as LC3-II and Beclin-1.

ACNs exhibit a dual role in regulating ROS levels—providing protection in OP, RA, and OA by reducing ROS levels, while exerting anticancer effects in OS by elevating ROS levels—due to differences in cellular redox sensitivity, target cell types, and activated signaling pathways across various environments. Under physiological or pathological conditions, when oxidative stress is excessive (e.g., in OP, RA, and OA), ACNs primarily act as antioxidants to mitigate ROS-induced damage. This suggests that in normal or dysfunctional bone/cartilage cells (osteoblasts, chondrocytes), ACNs scavenge excess ROS to restore redox balance, as these cells are highly sensitive to oxidative stress-induced dysfunction or death. Conversely, in OS, ACNs exploit the altered redox tolerance of cancer cells to elevate ROS levels above lethal thresholds, thereby triggering apoptosis and autophagic cell death. This mechanism aligns with observations in RA-FLS, where LRAC selectively induce ROS accumulation via NOX4, activating the NLRP3/IL-1β/caspase-1 pathway to promote apoptosis in proliferating synovial fibroblasts while protecting normal immune cells. OS cells have disrupted redox metabolism, resulting in a narrower ROS tolerance range compared to normal cells. ACNs disrupt this balance by enhancing ROS production (e.g., activating NADPH oxidase or causing mitochondrial dysfunction) while simultaneously inhibiting the antioxidant defense mechanisms of tumor cells. This dual action—elevating ROS levels to cytotoxic thresholds while blocking adaptive antioxidant responses—drives apoptosis and autophagy, consistent with the ROS-dependent anticancer effects observed with other natural compounds. Crucially, this dual action is mediated through cell type-specific signaling pathways. In normal bone cells, ACNs activate pro-survival pathways (e.g., SIRT1 in osteoblasts, PI3K/Akt in pre-osteoblasts, and Nrf2/NF-κB in chondrocytes) to counteract ROS. Conversely, in overproliferating or malignant cells, they exploit intrinsic redox vulnerabilities to induce cell death mechanisms such as apoptosis and autophagy. Thus, the ROS-regulating effects of ACNs are not inherently pro-oxidative or antioxidant but are contextually adjusted, making them a versatile therapeutic agent for treating various bone-related diseases.

Anthocyanins reduce the production of pro-inflammatory factors (such as TNF-α, IL-1β, and IL-6) by inhibiting the NF-κB and MAPK signaling pathways, while enhancing the antioxidant capacity of chondrocytes to protect the joint matrix from oxidative damage. They also maintain bone balance by inhibiting osteoclast formation and activity, and promoting osteoblast differentiation and mineralization. For osteosarcoma, *in vitro* studies indicate that certain anthocyanins have the potential to induce cancer cell apoptosis, inhibit proliferation, and reduce migration. NSAIDs primarily block the production of prostaglandins and thromboxane A by inhibiting COX enzymes (especially COX-2), thereby rapidly alleviating pain and inflammation. However, they offer no protective effect against cartilage degradation and may cause gastrointestinal, renal, and cardiovascular adverse reactions with long-term use (166, 167) Intra-articular injection of corticosteroids for osteoarthritis remains controversial. Current evidence suggests that this therapy not only lacks therapeutic benefits but may also exacerbate joint damage in the long term (168, 169). Biologics target specific inflammatory factors and are effective for rheumatoid arthritis (RA) but have limited efficacy for OA, along with risks of infection and high costs (170, 171). Therefore, although anthocyanins have a much weaker effect than NSAIDs for rapid pain relief, corticosteroids for potent anti-inflammatory effects, or biologics for treating RA and other diseases, their core value lies in their excellent antioxidant/anti-inflammatory properties and good safety profile. They are more suitable as part of a healthy lifestyle (dietary supplement) or as an adjunct to existing drug therapies.

Nevertheless, the limitations of the current study cannot be ignored. First, ACNs are inherently unstable due to their sensitivity to various physicochemical factors such as pH, temperature, oxygen and light, which significantly limits their practical applications. On the other hand, the bioavailability of ACNs is low, and their stability and absorption efficiency in the digestive tract remain important factors limiting their therapeutic effects. Future research needs to focus on the precise targeting of ACNs to ensure that they can reach the site of action directly while reducing degradation. Since ACNs and their derivatives have significant differences in bioavailability, further in-depth studies on the bioavailability characteristics of different derivatives can be conducted to achieve a more efficient performance of their biological functions and clinical applications. Secondly, although the role of ACNs in MSDs has been fully verified in animal model studies and basic experiments, their effectiveness and safety in different populations need to be further confirmed by clinical trials. In the future, multicenter, large-sample, randomized controlled trials should be conducted to clarify the optimal dosage of ACNs, the range of indications, and possible adverse effects. Finally, the synergistic effects of ACNs in combination therapy with conventional drugs have not been fully explored. Future studies could focus on examining whether ACNs can enhance the efficacy of NSAIDs, antirheumatic drugs, or bone resorption inhibitors while reducing their side effects. Overall, ACNs, with their wide range of sources and low toxicity profile, provide a valuable scientific basis for the development of novel phytochemicals targeting MSDs. However, clinical studies on ACNs for the treatment of MSDs are still limited, and challenges such as improving stability and bioavailability, and clarifying clinical efficacy and safety still need to be overcome in the transition from laboratory research to clinical application, which is expected to become an important tool for the treatment of MSDs in the future.

## Glossary

MSDs: Musculoskeletal disorders
ACNs: Anthocyanins
OP: Osteoporosis
RA: Rheumatoid arthritis
OA: Osteoarthritis
OS: Osteosarcoma
NSAIDs: Nonsteroidal anti-inflammatory drugs
pH: Potential of hydrogen
RANKL: Receptor activator of nuclear factor-kappaΒ ligand
RANK: Receptor activator of nuclear factor-kappaΒ
sRANKL: Soluble RANKL
OVX: Ovariectomized
BV/TV: Ratio of bone volume to tissue volume
Tb.Th: Trabecular thickness
Tb.N: Trabecular number
N.Nd/N.Tm: Node to terminus ratio
Tb.Sp: Trabecular separation
Ct.Th: Cortical thickness
SMI: Structure model index
ORAC: Oxygen radical absorbance capacity
CT: Computed tomography
NF-κB: Nuclear factor-kappaΒ
CTX-1: C-terminal telopeptide of type I collagen
CAT: Catalase
TNF-α: Tumor necrosis factor-alpha
P: Phosphorus
Ca: Calcium
Runx2: Runt-related transcription factor 2
OCN: Osteocalcin
OPN: Osteopontin
BSP: Bone sialoprotein
BMP: Bone morphogenetic protein
Smad: Small mothers against decapentaplegic
GSK-3β: Glycogen synthase kinase-3 beta
Sirt1: Sirtuin 1
ALP: Alkaline phosphatase
Osx: Osterix
Mepe: Matrix extracellular phosphoglycoprotein
Col1α1: Collagen type I alpha 1 chain
OPG: Osteoprotegerin
t-BHP: Tert-butyl hydroperoxide
EFSA: European Food Safety Authority
ADI: Acceptable daily intake
NFATc1: Nuclear factor of activated T cells calcineurin-dependent 1
DC-STAMP: Dendritic cell-specific transmembrane protein
OC-STAMP: Osteoclast stimulatory transmembrane protein
CTR: Calcitonin receptor
CtsK: Cathepsin K
SOD: Superoxide dismutase
GPX: Glutathione peroxidase
ROS: Reactive oxygen species
BSO: Butionine sulfoximine
MDA: Malondialdehyde
IL-1β: Interleukin-1 beta
COX-2: Cyclooxygenase-2
iNOS: Inducible nitric oxide synthase
NO: Nitric oxide
AIA: Adjuvant-induced arthritis
CIA: Collagen-induced arthritis
MAPKs: Mitogen-activated protein kinases
ERK: Extracellular signal-regulated kinase
JNK: c-Jun N-terminal kinase
FLS: Fibroblast-like synoviocytes
IL-17A: Interleukin-17A
IL-17RA: IL-17 receptor subunit A
Cyr61: Cysteine-rich angiogenic inducer 61
IL-23: Interleukin-23
PI3K/Akt: Phosphoinositide-3-kinase/protein kinase B
GM-CSF: Granulocyte-macrophage colony-stimulating factor
TLR3: Toll-like receptor 3
IL-4: Interleukin-4
IL-6: Interleukin-6
IL-10: Interleukin-10
IL-18: Interleukin-18
IL-21: Interleukin-21
FoxP3: Forkhead box P3
ROR-γT: Retinoic acid-related orphan receptor gamma t
MMPs: Matrix metalloproteinases
Treg: Regulatory T
NK: Natural killer
Sirt6: Sirtuin 6
Tfh: T-follicular helper
Tfr: T follicular regulatory
PGE2: Prostaglandin E2
STAT: Signal transducer and activator of transcription
Th17: T helper 17
H₂O₂: Hydrogen peroxide
ONOO^−^: Peroxynitrite
T-AOC: Total antioxidant capacity
NOX4: NADPH oxidase 4
NLRP3: NLR family pyrin domain containing 3
IκB: Inhibitor of kappa B
p-p65: Phosphorylated p65
PIAS3: Protein inhibitor of activated STAT-3
DMM: Destabilization of the medial meniscus
MIA: Monosodium iodoacetate
NKG2D: Natural killer group 2D
ECM: Extracellular matrix
AGEs: Advanced glycation end products
Nrf2: Nuclear factor (erythroid-derived 2)-like 2
c-PARP: Cleaved poly (ADP-ribose) polymerase
NIK: NF-kB-inducing kinase
IRAK1: IL-1 receptor-associated kinase-1
IKK: IκB kinase
CII: Type II collagen
UA: Uronic acid
P21: Cyclin-dependent kinase inhibitor 1
s-GAG: Sulfated glycosaminoglycan
ADAMTS5: A disintegrin and metalloproteinase with thrombospondin motifs 5
HA: Hyaluronic acid
PPARγ: Peroxisome proliferator-activated receptor gamma
Bax: Bcl-2-associated X protein
Bcl-xl: B-cell lymphoma-extra large
AMPK: Adenosyl monophosphate-dependent protein kinase
FOXO3a: Forkhead box O3
Mv: Malvidin
Pg: Pelargonidin
Dp: Delphinidin
Pn: Peonidin
Pt: Petunidin
Cy: Cyanidin
EMT: Epithelial-to-mesenchymal transition
AEBR: Anthocyanin-rich extract from black rice
VME: *Vaccinium myrtillus* extract
MBE: Maqui berry extract
BBs: Blackberries
C3G: Cyanidin-3-glucoside
BC: Blackcurrant
AHs: Anthocyanin-enriched polyphenols from *Hibiscus syriacus* L.
PDS: Prednisolone
STZ: Streptozotocin
LPS: Lipopolysaccharide
DEXA: Dual-energy X-ray absorptiometry
BMD: Bone mineral density
OSM: Oncostatin M
MSCs: Esenchymal stem cells
BJ: Blueberry juice
D3R: Delphinidin-3-rutinoside
Malβg: Malvidin-3-O-β glucoside
GPE: Grape polyphenol extract
PR: Propolis
Pg-3-glc: Pelargonidin-3-O-glucoside
Wnt: Wingless-related integration site
HAC: Human articular chondrocytes
PINP: N-terminal propeptide of type I procollagen
TRACP 5b: Tartrate-resistant acid phosphatase 5b
TRAP: Tartrate-resistant acid phosphatase
Atp6v0d2: ATPase H^+^ transporting V0 subunit d2
Tm7sf4: Transmembrane 7 subfamily member 4
Acp5: Acid phosphatase 5
Bglap: Bone gamma carboxyglutamate protein
IFN-γ: Interferon-gamma
IgG: Immunoglobulin G
ROCK: Rho-associated coiled-coil-containing kinase
MIP1α: Macrophage inflammatory protein-1 alpha
Drp-1: Dynamin related protein 1
PD-L1: Programmed death ligand-1
ADAM17: A disintegrin and metalloproteinase domain 17
PBLs: Peripheral blood-derived mononuclear leukocytes
RFs: Rheumatoid factors
HIF-1α: Hypoxia-inducible factor 1 alpha
CXCR4: C-X-C chemokine receptor type 4
PBMCs: Peripheral blood mononuclear cells
BMMs: Bone marrow macrophages
MNCs: Mononuclear cells
MDM: Monocytes-derived macrophages
HSP27: Heat shock protein 27
CCR7: CC-chemokine receptor-7
HC: Hydrocortisone
AEBS: Anthocyanin extracted from black soybean seed coats
RRE: Red raspberry extract
ME: Mulberry fruit extract
LRAC: *L. ruthenicum* anthocyanins
PCA: Protocatechuic acid
P3G: Peonidin-3-O-glucoside
CC: Cyanidin chloride
PC: Peonidin chloride
